# Induction of HIV‐Specific T Cell Responses Using αDC1 Pulsed With Conserved HIV‐1 Peptides

**DOI:** 10.1155/jimr/5457670

**Published:** 2026-05-15

**Authors:** Laís Teodoro Da Silva, Marina Mazzilli Ortega, Silvia de Jesus Mota, Guilherme Castellani, Dennyson Leandro M. Fonseca, Larissa Danielle Bahls Pinto, Gabriela Justamante Handel Schmitz, Isabela Leite, Theo Leite, Mariana Amelia Monteiro, Sandra Marcia Muxel, Robbie B. Mailliard, Alberto José da Silva Duarte, Telma Miyuki Oshiro

**Affiliations:** ^1^ Laboratory of Medical Investigation in Dermatology and Immunodeficiencies (LIM-56), Hospital das Clinicas HCFMUSP, School of Medicine, University of Sao Paulo, Sao Paulo, Brazil, usp.br; ^2^ Laboratory of Molecular Immunology Research miRlab, School of Arts, Science and Humanities (EACH), University of Sao Paulo, Sao Paulo, Brazil, usp.br; ^3^ Department of Clinical Analysis and Biomedicine (DAB), Postgraduate Program in Biosciences and Physiopathology (PBF), Maringá, Brazil; ^4^ Department of Basic Health Sciences (DBS), Laboratory of Immunogenetics at the State University of Maringá (Imunogen-UEM), Maringá, Brazil; ^5^ Postgraduate Program on Immunology, Institute of Biomedical Science (ICB), University of Sao Paulo (USP), Sao Paulo, São Paulo, Brazil, usp.br; ^6^ Department of Medicine, Division of Infectious Diseases, University of Pittsburgh School of Medicine, Pittsburgh, 15261, Pennsylvania, USA, pitt.edu

## Abstract

Despite the effectiveness of antiretroviral therapy (ART) in suppressing HIV‐1 replication and reducing morbidity, viral eradication remains unachievable due to the persistence of latent reservoirs, particularly within memory CD4^+^ T cells. Dendritic cell (DC)‐based immunotherapeutic approaches have emerged as potential strategies to enhance antigen‐specific immune responses against HIV‐1. In this context, alpha‐type‐1 polarized DCs (αDC1) are notable for their capacity to produce interleukin‐12p70 (IL‐12p70) and induce robust Th1 and cytotoxic T lymphocyte (CTL) responses. However, clinical outcomes have been inconsistent, often influenced by host factors and methodological variability in DC generation and antigen delivery. The high genetic diversity of HIV poses challenges for vaccine development, whereas conserved regions such as gag and pol represent promising immunogenic targets. The present study aimed to evaluate a set of previously identified conserved HIV‐1 peptides for their ability to stimulate HIV‐specific T cell responses when presented by αDC1 in vitro in coculture assays using samples from a Brazilian cohort, as part of a preparatory phase for a clinical trial. For this purpose, monocytes were differentiated into αDC1 using IL‐4 and GM‐CSF. On Day 5, the cells were pulsed with 14–21mer HIV‐1 peptide pools (gag and pol) and matured by the addition of IFN‐α, IFN‐γ, IL‐1β, and TNF‐α. After 48 h, αDC1 were harvested and cocultured with autologous T cells for 6 or 20 days. Pulsed‐αDC1 were able to induce immune responses through the production of IFN‐γ in T cells, with the subpopulation of effector memory CD4^+^ T cells (*T*_EM_) being responsible for the production of this cytokine after reexposure to the HIV antigens.

## 1. Introduction

More than 40 years after its identification, HIV/AIDS remains a global pandemic. Although the introduction of highly active antiretroviral therapy (HAART) has led to a significant reduction in morbidity and mortality among people living with HIV/AIDS (PLWHA), mainly due to the suppression of plasma viral load, this approach does not provide a definitive cure [1, 2]. One of the main challenges is the persistence of latent viral reservoirs, especially within CD4+ T memory cells, which remains feasible even after years of successful viral suppression [3]. While effectively controlling viral replication in most individuals, treatment interruption commonly leads to a rapid viral rebound, highlighting the critical need for complementary therapeutic strategies aimed at reducing latent reservoirs and/or enhancing the antiviral immune response [4, 5].

In this context, strategies based on dendritic cells (DC) have been increasingly explored, once these cells activate adaptive immunity and show potential to induce specific responses against HIV‐1 [6]. DCs are the principal antigen presentation cells in immunological systems that play a key role in the initiation and regulation of the immune response, linking innate and adaptive immunity [7, 8], and they are extremely important to activate and differentiate naive T cells into other T cell subtypes depending on the stimuli and signals received by them [9]. In HIV infection, several clinical trials using DCs as adjuvants have shown different outcomes. Some of them observed a transient reduction in patient’s viral load [10–16]. Nonetheless, other DC‐based interventions, including those employing viral mRNA or HIV‐derived peptides, have not demonstrated significant reductions in plasma viremia or prolonged viral suppression after antiretroviral therapy (ART) discontinuation [17–23]. Variations and limitations observed in these earlier DC studies can be attributed to multiple biological and technical factors, including host‐related variables, such as disease progression, human leukocyte antigen (HLA) genotype, and basal immune profile. However, other aspects related to the method of DC generation and maturation strategies used that affect the functional characteristics of the DC must also be considered. In addition, the methodology used to measure the efficacy of the immune response generated by DCs is equally important.

Regarding the methods of DC generation for clinical application, the protocols used to date have shown considerable variability, with differences in media composition, culture duration, cytokine and activation factor combinations used, and antigen delivery strategies implemented. Any of these parameters can influence the immunogenicity and immunotherapeutic potential of the DC product, affecting not only the expression of critical co‐stimulatory molecules and key cytokines but also the efficiency of antigen presentation and DC trafficking to lymph nodes (revised by [24]). Protocols to optimize DC programming for their improved ability to drive effective Th1‐biased cellular immune responses, the type of response desirable for antiviral immunity, have been widely explored. In this regard, the alpha‐type‐1 polarized DC (αDC1) platform was developed for the purpose of achieving a DC with a mature status having a higher capacity to produce interleukin‐12p70 (IL‐12p70), a key cytokine involved in the induction of Th1 and cytotoxic T lymphocyte (CTL) responses, which are essential effector cells for fighting intracellular pathogens and viruses such as HIV‐1 [25].

Dealing with viral heterogeneity, adaptation, and immune escape are also major factors to overcome when considering antigen selection in the designing of a DC‐based therapeutic approach to control HIV infection. The high genetic variability of HIV, especially in the codifier regions of envelope (env) (gp120/gp41), has been a major challenge for the development of effective prophylactic and therapeutic interventions [26, 27]. Many strategies have been explored to address this point, including use of pooled antigenic peptides to elicit broadly reactive polyfunctional T cell responses against regions of Gag and Pol having less variability and more functional constraints [28], as well as implementation of mRNA delivery to DCs for their endogenous expression and presentation of Tat‐, Rev‐, and Nef‐associated peptides [21]. The identification and utilization of highly conserved and immunogenic peptides have demonstrated the potential to induce specific cellular responses, capable of overcoming the challenge of viral heterogeneity, while redirecting or focusing CTL responses toward more critical fitness‐constrained regions of HIV [29]. For that purpose, Garcia‐Bates et al. [29] observed that proinflammatory DCs loaded with longer HIV‐conserved peptides (afferent stimulation) promote processing and presentation of HIV‐specific MHC II‐restricted peptides for CD4^+^ T cell responses as well as the cross‐presentation of exogenous antigen in the context of MHC class I molecules, leading to efficient induction of primary CTL responses. Upon a secondary restimulation (efferent) with shorter peptides derived from these larger peptides (i.e., contained within the longer sequences), it was shown that MHC class I‐restricted CTL responses toward such fitness‐constrained regions of the virus could indeed be effectively revealed.

Once these immunologically relevant, ultraconserved, and topologically constrained HIV‐1 peptides were identified and successfully tested in a Thai cohort study [29], and considering that this afferent induction of T cell immune responses covers a broad range of possible HLA‐associated haplotypes, we aimed to determine if this strategy could be universally applied with similar success by utilizing samples from a Brazilian cohort. Importantly, the Brazilian population is composed of a wide mixture of many ethnic groups, predominantly Caucasians, Blacks, Indians, and the mixed‐race descendants of these ethnic groups, thus composing one of the most heterogeneous populations in the world [30, 31]. In this present study, we loaded αDC1 with these selected HIV‐1 peptides and tested them in preliminary in vitro assays conducted within the framework of a forthcoming clinical trial, aiming to evaluate their capacity to induce primary HIV‐specific T cell responses and to reactivate HIV‐specific memory T cells.

## 2. Methods

### 2.1. Samples Collection

Heparin blood samples obtained from 23 PLWH receiving HAART were used in the present study. For inclusion all participants had maintained undetectable plasma HIV‐1 RNA viral load levels for at least 6 months prior to enrollment, CD4^+^ T cell count ≧500 cells/mm^3^, and had no history of opportunistic infections, chronic diseases, immune dysfunction, or AIDS‐defining events. All the subjects were recruited at the ADEE—3002 Clinic of the Dermatology Department of the Hospital das Clınicas, Sao Paulo, Brazil. Written informed consent was obtained according to the protocols of the Hospital das Clinicas Ethical Committee (CAPPesq; Sao Paulo, Brazil) under approval protocol #6.020.101. All the participants gave informed consent at the time of recruitment for the study.

### 2.2. HLA Genotyping

Genomic DNA was isolated from the donor buffy coat blood products using the QIAamp DNA Blood Mini Kit (Qiagen ‐ Hilden, Germany) according to the manufacturer’s instructions. HLA typing was performed via next‐generation sequencing on the iSeq 100 platform (Illumina ‐ San Diego, United States) using the Alltype FASTPlex NGS 11 Loci kit (One Lambda Thermo Fisher ‐ Los Angeles, United States). Sequencing data was analyzed using TypeStream Visual 3.2 software (One Lambda, Thermo Fisher ‐ Los Angeles, United States).

### 2.3. HIV Peptides Selection in Peripheral Blood Mononuclear Cells (PBMCs)

PBMCs were obtained by centrifugation using a Ficoll‐Hypaque gradient, and cell concentration was adjusted to 1 × 10^6^ cells/mL in a 48‐well plate. PBMCs were stimulated individually with 1 µg/mL HIV‐1 peptide of 14–21–amino acids length (GenScript), described on Table 1. Those peptides were previously identified by Garcia‐Bates et al. [29] using antigen prediction tools that consider ultraconserved and topologically constrained HIV‐1‐associated epitopes. IL‐2 (20 UI/mL) and IL‐7 (10 ng/mL) (PeproTech), were added on days 1 and 4. On Day 6, the cells were exposed again to the peptides for 2 h, and, after this period, the cells received 20 μg/mL of Brefeldin A (Sigma–Aldrich). After 18 h, cells were harvested and stained with specific monoclonal antibodies for the detection of IFN‐γ production by CD4+ CD8^+^ T cells.

**Table 1:** Pools of selected overlapping HIV peptides for the Gag and Pol regions.

Peptide	Sequences
Gag 15	WVKVVEEKGFNPEVIPMFSAL
Gag 17	EGATPQDLNMMLNIVGGHQAA
Gag 21	GPIPPGQMREPRGSDIAGTTS
Gag 22	RGSDIAGTTSTLQEQIGWMTN
Gag 27	QGPKEPFRDYVDRFYKTLRAE
Gag 34	AEAMSQAQHANIMMQRGNFKG
Gag 35	IMMQRGNFKGQKRIKCFNCGK
Pol 22	ILVAVHVASGYIEAEVIPAET
Pol 32	FTIPSINNETPGIRYQYNVLP

### 2.4. αDC1 Obtained

After selection of HIV‐1 peptides using PBMCs, peptide‐pulsed αDC1 were used to induce immune responses in autologous T cells. For this purpose, monocytes were isolated from PBMCs using plastic adherence [32]. Briefly, PBMCs were maintained for 2 h in flasks at a concentration of 5 × 10^6^/mL in RPMI 1640 medium (Thermo Fisher Scientific) at 37°C and 5% CO_2_. After incubation, nonadherent cells were removed by washing and cryopreserved to be used in the following assays, and adherent cells, predominantly monocytes, were cultured in AIM‐V serum‐free medium (Thermo Fisher Scientific) supplemented with 100 ng/mL recombinant human GM‐CSF and 200 ng/mL recombinant human IL‐4 (PeproTech) for generation of immature DCs (iDCs), following the protocol described by Mailliard et al. [25], with modifications.

On Day 5, iDCs were pulsed with 14–21mer HIV‐1 peptide pools (Gag 15, Gag17, Gag27, Gag 34, Gag 35, Pol‐net‐2, and Pol.14.a), at a final concentration of 1 µg/mL. After 2 h of incubation, iDCs were activated using a proinflammatory cocktail containing 1000 UI/mL IFN‐α 2 b (Miltenyi Biotec), 1000 UI/mL IFN‐γ (PeproTech), 10 ng/mL IL‐1β (PeproTech), and 25 ng/mL TNF‐α (Peprotech). On Day 7, mature αDC1 were harvested to be used on the following assay.

For quality control purposes, some of the cells were phenotypically and functionally characterized. αDC1 exhibited the classic phenotypic and functional features with augmented expression of maturation markers and high IL‐12p70 production, demonstrating that the differentiation and maturation protocol successfully reproduced the expected αDC1 profile [25] (Supporting Information 1: Figure S1A, B). Also, despite monocyte isolation being performed by adhesion rather than purification using CD14+ magnetic beads, we observed that the phenotypic profile of αDC1 cells was similar between both protocols (Supporting Information 2: Figure S2A–C), endorsing their use in the present study.

### 2.5. Autologous T Cells Activation by αDC1

To assess the capacity of αDC1 to activate autologous T cells, two sequential coculture protocols were conducted: the first spanning 6 days and the second extending over 20 days. Initially, αDC1 were cocultured with previously thawed autologous nonadherent cells at a ratio of 1 αDC1 to five nonadherent cells. Unstimulated T cells were used as a negative control and for measurement of basal IFN‐γ production. The positive control was composed of lymphocytes stimulated with Phorbol 12‐myristate 13‐acetate (PMA, 30 ng/mL; Sigma–Aldrich) and ionomycin (0,3 μg/mL; Sigma–Aldrich) in the last 18 h of incubation.

Based on the protocol described by Zelba et al. [33], with modifications, the short‐term protocol was performed firstly added cells in the 48‐well‐plates and incubated at 37°C in an atmosphere containing 5% CO_2_ for 6 days in the presence of IL‐2 (20 UI/mL) and IL‐7 (10 ng/mL) (PeproTech), with the cells receiving fresh complete culture medium on Day 3. For the last 18 h in culture, cells were restimulated with HIV peptide pools, and after 2 h, 20 μg/mL of Brefeldin A (BFA, Sigma–Aldrich) was added to block any further protein transport from the endoplasmic reticulum to the Golgi apparatus. After incubation, T cell activation was assessed through IFN‐y production by flow cytometry.

Alternatively, aiming to investigate the potential of αDC1 to prime memory T cells responses, a longer coculture assay, following the protocol described by Kurle et al. [34], with modifications, was conducted. To this protocol, αDC1 were cocultured with previously thawed autologous nonadherent cells at a ratio of 1 αDC1 to 5 nonadherent cells during 20 days in the presence of IL‐2 (20 UI/mL) and IL‐7 (10 ng/mL), with the cells receiving fresh complete culture medium on Days 5, 10, and 15. In order to assess HIV‐specific memory T cell response, on Day 19, cells were reexposed to the peptide pools for 2 h. Alternatively, to detect memory response specifically of TCD8 cells, some wells were restimulated using shorter 8–11mer HIV‐1 epitopes (G15c, G17d, G27a, G34a, P22b, P32b, and P32c; Supporting Information 3: Table S1) contained within 14–21mer peptide pools.

To verify T cell immunophenotyping, after this period, some wells received 20 μg/mL of BFA in the last 18 h of incubation. Finally, on Day 20, part of these cells was harvested for analysis by flow cytometry, while the remaining cells were used to evaluate their cytotoxic potential.

### 2.6. Cell Staining for Flow Cytometry Analysis

For the selection of HIV peptides in PBMCs, T cells were stained with BD Horizon Fixable Viability Stain to determine cell viability; and with the surface markers CD3 PE‐Cy7 (clone UCHT1), CD4 BV605 (clone RPA‐T4), CD8 PE (clone HIT8α), and the intracellular marker IFN‐γ PerCP‐Cy 5.5 (clone B27; BD Biosciences) using the Cytofix/Cytoperm kit (BD Bioscience) as recommended by the manufacturer.

To assess IFN‐γ production, cytotoxicity, and memory in αDC1‐sensitized T cells, in addition to the monoclonal antibodies listed above, cells were stained with the following surface markers: CCR7 Alexa Fluor 700 (clone 150503), CD45RA FITC (clone HI100), and Granzyme (GzB) B V450 (clone GB11; all from BD Biosciences).

The cells were acquired on an LSR Fortessa using DIVA software, and analysis was performed with FlowJo vX v10.10 (Becton, Dickinson and Company) and GraphPad Prism v.8 software (GraphPad Software Inc., CA, USA). Specific responses were reported as the mean of duplicate samples. The analysis strategies applied to the analyzed T cell populations are depicted in Supporting Information 4: Figure S3.

### 2.7. Statistical Analysis

Statistical analyses were performed using GraphPad Prism v.8 software (Graph‐Pad Software) and the R programming language (version R version 4.5.0). Normality of the data was assessed using the Shapiro–Wilk test in combination with Q–Q plots. Variables with Shapiro–Wilk *p*‐values < 0.05 were considered to follow an asymmetric distribution (Supporting Information 5: Table S2, Supporting Information 6: Table S3 and Supporting Information 7: Table S4). In this case, differences between two groups or multiple comparisons were evaluated using the Wilcoxon–Mann–Whitney test. Statistical significance was defined as a two‐sided *p*‐value < 0.05 and false discovery rate (FDR) < 0.05 for multiple comparisons (Supporting Information 8: Table S5, Supporting Information 9: Table S6 and Supporting Information 10: Table S7). Furthermore, effect size was calculated using the Wilcoxon rank‐based effect size (*r*) from the rstatix R package), with confidence intervals (95%) computed to assess the magnitude of the observed effects [35]. The magnitude of the effect was interpreted as small (*r* ≥ 0.1), moderate (*r* ≥ 0.3), or large (*r* ≥ 0.5) (Supporting Information 11: Figure S4 and Supporting Information 12: Figure S5). In addition, correlations among variables were established using Spearman’s correlation (*pho* > 0.7 and *p* < 0.05; Supporting Information 13: Figure S6).

## 3. Results

The characteristics of the recruited patients are described in Table 2. All patients were male; under suppressive HAART for at least 7 years and an undetectable viral load for more than 3 years. High‐resolution HLA genotyping was performed for the major classical HLA class I and class II loci. Age varies from 26 to 62 years, with a median age of 53 years; time since diagnosis in years varies from 9 to 33 years (median of 14 years); and the nadir CD4^+^ T cell counts vary from 7 to 773 cells/mm^3^ (median of 509 cells/mm^3^).

**Table 2:** Demographic and clinical characteristics of study volunteers.

Patient ID	Age (years)	Time from HIV diagnosis to study entry (years)	CD4+ T count (cells/µL)		HAART Regimen	HLA haplotype							
			Nadir	Basal		HLA‐A	HLA‐B	HLA‐C	HLA‐DRB1	HLA‐DQA1	HLA‐DQB1	HLA‐DPA1	HLA‐DPB1
P01	34	12	246	882	TDF + 3TC + DTG	ND	58:01:01/ 27:05:02	07:18:01/ 02:02:02	16:01:01/ 04:01:01	03:03:01/ 01:02:02	05:02:01/ 03:02:01	01:03:01/ 01:03:01	ND
P02	57	12	237	502	RTV + DRV + DTG	25:01:01/ 23:01:01	53:01:01/ 18:01:01	12:03/ 06:02	14:54/ 13:02	ND	06:04:01/ 05:03:01	01:03:01/ 01:03:01	03:01:01/ 03:01:01
P03	59	10	344	829	3TC + DTG	26:01:01/ 25:01:01	38:01:01/ 18:01:01	12:03:01/ 12:03:01	15:01:01/ 13:01:01	01:03:01/ 01:02:01	06:03:01/ 06:02:01	02:02:02/ 01:03:01	ND
P04	62	26	25	564	3TC + DTG	33:01:01/ 02:01:01	44:02:01/ 14:02:01	08:02:01/ 05:01:01	15:01:01/ 13:01:01	01:03:01/ 01:02:01	06:03:01/ 06:02:01	01:03:01/ 01:03:01	04:01:01/ 04:01:01
P05	51	19	183	564	3TC + DTG	02:01:01/ 01:01:01	49:01:01/ 14:02:01	07:01:01/ 02:02:02	13:02:01/ 01:02:01	01:02:01/ 01:01:02	06:04:01/ 05:01:01	02:01:01/ 01:03:01	ND
P06	57	13	628	1693	3TC + DTG	29:02:01/ 23:01:01	44:03:01/ 44:03:01	16:01:01/ 04:01:01	08:01:01/ 07:01:01	04:01:01/ 02:01:01	04:02:01/ 02:02:01	01:03:01/ 01:03:01	04:01:01/ 04:01:01
P07	49	24	530	911	3TC + DTG	03:01:01/ 02:01:01	35:08:01/ 18:01:01	04:01:01/ 02:10:01	15:03:01/ 10:01:01	03:03:01/ 01:05:01	05:01:01/ 02:02:01	03:01/ 01:03	ND
P08	59	31	220	560	3TC + DTG	25:01:01/ 01:01:01	18:01:01/ 08:01:01	12:03:01 / 07:01:01	15:01:01/ 03:01:01	05:01:01/ 01:02:01	06:02:01/ 02:01:01	02:01:02/ 01:03:01	23:01:01/ 01:01:01
P09	59	17	246	812	3TC + DTG	03:01:01/ 02:01:01	49:01:01/ 40:01:02	07:02:01/ 03:04:01	13:02:01/ 11:02:01	05:05:01/ 01:02:01	06:04:01/ 03:19:01	01:03:01/ 01:03:01	04:01:01/ 04:01:01
P10	38	9	363	557	3TC + DTG	32:01:01/ 31:01:02	52:01:01/ 27:05:02	15:02:01/ 12:02:02	15:02:01/ 03:01:01	01:02:01/ 02:02:01	06:01:01/ 02:01:01	02:01:01/ 01:03:01	ND
P11	59	15	7	888	3TC + DTG	03:01:01/ 02:01:01	49:01:01/ 40:01:02	07:02:01/ 03:04:01	13:02:01/ 11:02:01	05:05:01/ 01:02:01	06:04:01/ 03:19:01	01:03:01/ 01:03:01	04:01:01/ 04:01:01
P12	35	9	661	837	TDF + 3TC + DTG	29:02:01/ 01:01:01	44:03:01/ 38:01:01	16:01:01/ 12:03:01	16:01:01/ 07:01:01	02:01:01/ 01:01:01	05:02:01/ 02:02:01	01:03:01/ 01:03:01	04:01:01/ 04:01:01
P13	41	14	509	920	RTV + DRV + DTG	02:05:01/ 02:01:01	41:01:01/ 13:02:01	07:01:01/ 06:02:01	13:05:01/ 07:01:01	05:05:01/ 02:01:01	03:01:01/ 02:02:01	02:01:01/ 01:03:01	ND
P14	26	7	568	1249	TDF + 3TC + DTG	33:01:01/ 02:01:01	44:02:01/ 14:02:01	08:02:01/ 05:01:01	15:01:01/ 13:01:01	01:03:01/ 01:02:01	06:03:01/ 06:02:01	01:03:01/ 01:03:01	04:01:01/ 04:01:01
P15	39	18	253	878	TDF + 3TC + DTG	29:02:01/ 02:01:01	39:01:01/ 07:02/01	12:03:01/ 07:02:01	16:01:01/ 15:01:01	01:02:02/ 01:02:01	06:02:01/ 05:02:01	01:03:01/ 01:03:01	ND
P16	51	33	485	1384	ABC + 3TC + DRV + RTV	30:02:01/ 02:01:01	44:03:01/ 35:08:01	16:01:01/ 04:01:01	11:01:01/ 07:01:01	05:05:01/ 02:01:01	03:01:01/ 02:02:01	01:03:01/ 01:03:01	04:01:01/ 04:01:01
P17	61	9	664	917	3TC + DTG	23:01:01/ 01:01:01	57:01:01/ 44:03:01	06:02:01/ 04:01:01	16:01:01/ 13:03:01	05:05:01/ 01:02:02	05:02:01/ 03:01:01	01:03:01/ 01:03:01	124:01:02/ 04:01:01
P18	62	13	740	871	3TC + DTG	02:01:01/ 02:01:01	39:10:01/ 07:02:01	12:03:01/ 07:02:01	15:01:01/ 11:01:02	01:02:01/ 01:02:01	06:02:01/ 06:02:01	01:03:01/ 01:03:01	18:01:01/ 04:01:01
P19	53	12	720	1980	TDF + 3TC + DTG	24:02:01/ 01:01:01	52:01:01/ 14:02:01	12:02:02/ 08:02:01	ND/13:01	01:03:01/ 01:03:01	06:03:01/ 06:01:01	02:01:01/ 01:03:01	ND
P20	37	19	509	1369	TDF + 3TC + DTG	24:02:01/ 01:01:01	81:01:01/ 40:04:01	18:01:01/ 03:04:01	11:01:02/ 04:11:01	05:05:01/ 03:01:01	03:19:01/ 03:02:01	02:01:08/ 02:01:01	14:01:01/ 01:01:01
P21	59	28	668	927	TDF + 3TC + DTG	24:02:01/ 01:01:01	81:01:01/ 40:04:01	18:01:01/ 03:04:01	11:01:02/ 04:11:01	05:05:01/ 03:01:01	03:19:01/ 03:02:01	02:01:08/ 02:01:01	14:01:01/ 01:01:01
P22	46	14	539	1081	TDF + 3TC + DTG	30:04:01/ 11:01:01	58:02:01/ 35:01:01	06:02:01/ 04:01:01	15:03:01/ 01:01:01	01:02:01/ 01:01:01	06:02:01/ 05:01:01	03:01:01/ 02:01:01	105:01:01/ 11:01:01
P23	60	25	773	1285	3TC + DTG	68:02:01/ 01:01:01	53:01:01/ 15:10:01	04:01:01/ 03:04:02	04:11:01/ 03:01:01	05:01:01/ 03:01:01	03:02:01/ 02:01:01	01:03:01/ 01:03:01	ND
Median (25% and 75% IQR)	**53 (40–59)**	**14 (12–21, 5)**	**509 (246–644,5)**	**888 (820,5–1165)**	**N/A**	**N/A**	**N/A**	**N/A**	**N/A**	**N/A**	**N/A**	**N/A**	**N/A**

*Note*: Median frequency (and IQR, interquartile range: 25th to 75th percentile) for the age, time from HIV diagnosis to study entry, CD4^+^ T cells count, HAART regimen, and HLA haplotypes are shown. TDF, tenofovir; 3TC, lamivudine. Bold values indicate the median frequency (and IQR, interquartile range: 25th to 75th percentile) from each column.

Abbreviations: ABC, abacavir; DRV, darunavir; DTG, dolutegravir; N/A, nonapplicable; ND, not detected; RTV, ritonavir.

### 3.1. HIV‐Specific T Cell Immune Responses

**Figure 1:**
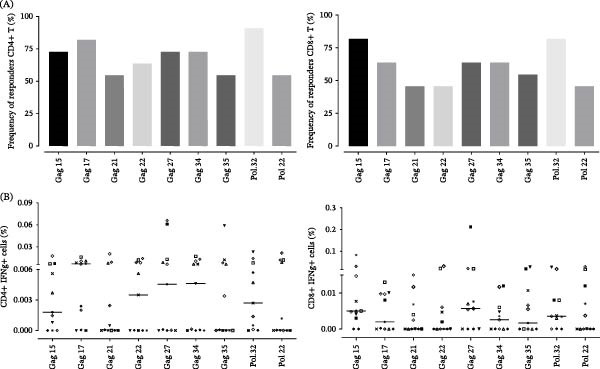
Breadth and magnitude of HIV‐specific T cell response. PBMCs were stimulated with HIV‐1 peptides and analyzed by flow cytometry. The frequency of positive peptide responses (A) and the percentage of IFN‐γ production (B) by CD4+ and CD8^+^ T cells are represented. Each symbol represents a studied individual. The horizontal lines indicate the medians of the studied conditions (*n* = 11).

**Figure 2:**
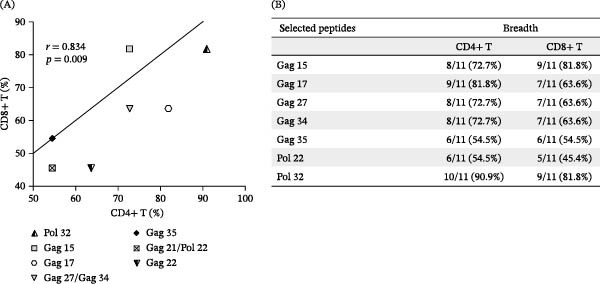
Peptides selection based on IFN‐γ production. PBMCs were stimulated with HIV‐1 peptides and IFN‐γ production was assessed by flow cytometry. In (A) correlation between the frequency of CD8+ and CD4^+^ T cells IFN‐γ production to all nine peptides tested (*p* < 0.01); and (B) breadth of T cell responses to all seven selected peptides. The correlation was established using Pearson’s correlation (*r* ^2^ > 0.95 and *p* < 0.05; *n* = 11).

Selection peptides were based on those who showed better results in terms of breadth and magnitude at the level of IFN‐γ production. As represented in Figure 1A, was observed to have a higher frequency of donors who responded to Pol 32 (≥81.8%), Gag 15 (≥72.7%), and Gag 17 (≥63.6%) for both, CD4+ and CD8^+^ T cells. Furthermore, all PLWH had responses to at least three of nine HIV‐1‐specific peptides (median = 11) used to stimulate PBMCs. When the percentage of IFN‐γ production by CD4+ and CD8+ T‐cells (magnitude) was taken into account, the greatest immune response was observed in CD4^+^ T cells to the peptides Gag 17, Gag 34, and Gag 27, while the highest percentage of HIV‐specific CD8^+^ T cells was observed for Gag 27, Gag 15, Pol 32, and Gag 34 (Figure 1B; Supporting Information 14: Table S8). As shown in Figure 2A, a positive correlation between the frequency of CD4+ and CD8^+^ T cell responses to Pol 32, Gag 15, Gag 17, Gag 27, and Gag 34 peptides (*p*  < 0.005) was observed. Finally, in addition to the Pol peptides, five of the seven Gag peptides that induced greater magnitude and breadth of T lymphocyte responses were selected to be used in the following tests, and are depicted in Figure 2B.

### 3.2. Pulsed αDC1 Promote IFN‐γ Production in CD4+ T Cells

**Figure 3:**
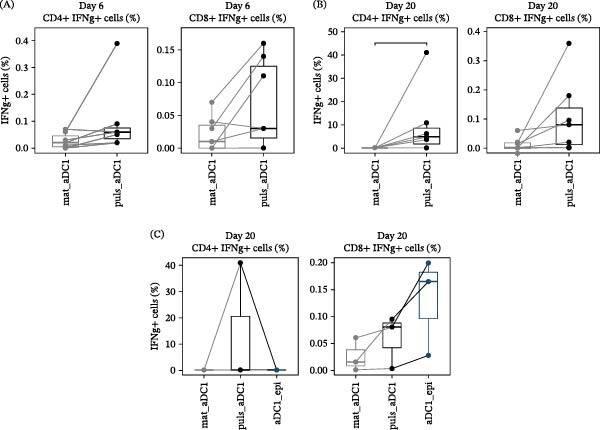
HIV‐specific T cell response induced by αDC1. T cells were cocultured with αDC1, and IFN‐γ production was measured by flow cytometry. The line graphs express individual results of IFN‐γ production by CD4+ and CD8^+^ T cells after 6 days (A, *n* = 6) or 20 days (B, *n* = 6) and in coculture with αDC1 (C, *n* = 3). T cells sensitized by mature αDC1 (mat_αDC1); αDC1 pulsed with HIV‐1 14–21mer peptide pools (puls_αDC1) and reestimulate on Day 19 using 14–21mer peptide pools; or αDC1 pulsed with HIV‐1 14–21mer peptide pools and restimulated with HIV‐1 8–11mer epitopes (puls_αDC1 + epitopes). The comparison of two variables was performed using the Mann–Whitney U test and the Wilcoxon test was used to compare three variables ( ^∗^ *p* < 0.05).

Initially, to verify whether the selected peptides were able to induce immune responses in αDC1‐sensitized T cells, αDC1 pulsed with 14–21mer HIV‐1 peptide pools were cocultured with autologous T cells for 6 days. The frequency of IFN‐y‐producing T cells is shown in Figure 3A, and the results obtained were subtracted from the negative control (unstimulated T cells). No statistically significant differences were observed between peptide‐pulsed and nonpulsed mature αDC1 in terms of IFN‐γ production for both CD4+ and CD8^+^ T cells.

**Figure 4:**
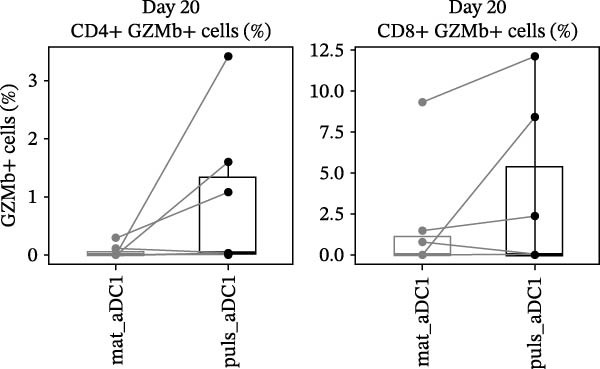
CTLs induced by αDC1. T cells were cocultured with αDC1 and granzyme B expression was assessed by flow cytometry. The line graphs express individual results of the percentage of granzyme B (GzB) produced by CD4+ (*n* = 5) and CD8+ (*n* = 4) T cells after 20 days in coculture with αDC1. T cells sensitized by mature αDC1 (mat_αDC1) or αDC1 pulsed with HIV‐1 14–21mer peptide pools (puls_αDC1) are shown.

Considering these results, another protocol was applied with the aim of inducing HIV‐1‐specific immune responses in T cells, in which αDC1 pulsed with 14–21mer HIV‐1 peptide pools were cocultured with autologous T cells for 20 days. IFN‐γ production was significantly higher by CD4^+^ T cells sensitized by pulsed αDC1 (4.8%) compared to mature αDC1, activated using a pro‐inflammatory cocktail without receiving HIV‐1 peptide pools (0.01%) (*p*  < 0.05; Figure 3B). It is possible to observe a greater production of IFN‐y by CD4^+^ T cells incubated with pulsed αDC1 for 20 days compared to 6 days, with an 80x increase in the secretion of this cytokine. Regarding CD8^+^ T cells, although we observed an increase in IFN‐γ production when cells were stimulated by 14–21mer‐pulsed αDC1, this increase did not reach statistical significance. In contrast, when CD8^+^ T cells were exposed to 8–11mer HIV‐1 epitopes on Day 19 of coculture, a significant increase in IFN‐γ production compared to mature αDC1 was observed ( ^∗^ *p*  < 0.05; Figure 3C). Concerning the potential of αDC1 pulsed with HIV‐1 peptides to induce CTLs, GzB, a key component of the perforin/GzB pathway, was studied. A tendency toward elevated levels of the cytotoxic marker was observed in lymphocytes incubated for 20 days with pulsed αDC1 compared with mature αDC1 but did not reach statistically significant differences (Figure 4).

### 3.3. HLA‐Association With T Cell Responses Triggered by DCs Stimulated With Afferents or Epitopes HIV‐1 Peptides

Peptide‐HLA ligation predictions were performed using the NetMHCpan EL algorithm available through the Immune Epitope DataBase (IEDB) platform. Peptides that were able to stimulate immune responses were subsequently found to display favorable predicted binding to at least one HLA allele expressed by responding individuals (Table 3), supporting the consistency of the observed responses. Several peptides demonstrated strong predicted binding to multiple HLA alleles, suggesting a potential presentation across the cohort. Nonetheless, the presence of the corresponding HLA allele did not uniformly result in detectable responses, highlighting that predicted binding affinity alone does not account for the activation of antigen‐specific T cells.

**Table 3:** Association between HIV‐1 peptides and HLA alleles.

Peptide	Peptide source	Sequence	Protein	Total responder	HLA+/total	#Respond/HLA+	HLA associations	Percentile rank (EL)
Gag.15	Afferent	**WVKVVEEKGFNPEVIPMFSAL**	Gag	5/7	0/7	0/7	—	—
G15c	Epitope	**EVIPMFSAL**	Gag	3/3	1/3, 1/3, 1/3, 1/3, 2/3, 2/3	1/1, 1/1, 1/1, 1/1, 2/2, 2/2	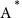 68:02, 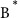 81:01, 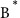 53:01, 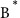 15:10, 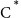 04:01, 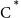 03:04	0.02/0.35/1.1/1.1/1.9/ 0.24
Gag.17	Afferent	**EGATPQDLNMMLNIVGGHQAA**	Gag	5/7	0/7	0/7	—	—
G17d	Epitope	**ATPQDLNMML**	Gag	3/3	1/3	1/1	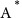 68:02, 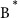 81:01, 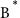 53:01, 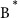 15:10	0.02/0.35/1.1 1.1
Gag.27	Afferent	**QGPKEPFRDYVDRFYKTLRAE**	Gag	5/7	1/7	1/1	DQA1 ^∗^01:01/DQB1 ^∗^02:02	1.6
G27a	Epitope	**FRDYVDRFY**	Gag	3/3	3/3, 1/3, 1/3, 2/3, 1/3	3/3, 1/1, 1/1, 2/2, 1/1	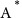 01:01, 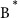 15:10, 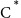 06:02, 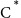 04:01, 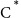 18:01	0.99/1.6/0.13/0.61/ 0.33
Gag.34	Afferent	**AEAMSQAQHANIMMQRGNFKG**	Gag	5/7	0/7	0/7	—	—
G34a	Epitope	**IMMQRGNFK**	Gag	3/3	0/3	0/0	—	—
Gag.35	Afferent	**IMMQRGNFKGQKRIKCFNCGK**	Gag	5/7	0/7	0/7	—	—
Pol.22	Afferent	**ILVAVHVASGYIEAEVIPAET**	Pol	5/7	2/7, 1/7, 1/7	2/2, 1/1, 1/1	DQA1 ^∗^03:01/DQB1 ^∗^03:02,DQA1 ^∗^05:01/DQB1 ^∗^03:02,DQA1 ^∗^03:01/DQB1 ^∗^02:01	0.4/0.25/1.3
P22b	Epitope	**HVASGYIEA**	Pol	3/3	1/3	1/1	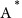 68:02	0.15
Pol.32	Afferent	**FTIPSINNETPGIRYQYNVLP**	Pol	5/7	0/7	0/7	—	—
P32b	Epitope	**NNETPGIRYQY**	Pol	3/3	3/3, 1/3	3/3, 1/1	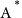 01:01, 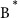 44:03	0.61
P32c	Epitope	**IRYQYNVL**	Pol	3/3	1/1	1/1	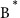 15:10, 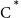 06:02	1.4/0.28

*Note:* Bold values represent the nucleotide sequences of each peptide.

### 3.4. IFN‐γ Production by Effector Memory CD4+ T Cells is Induced by Antigen Pulsed αDC1

**Figure 5:**
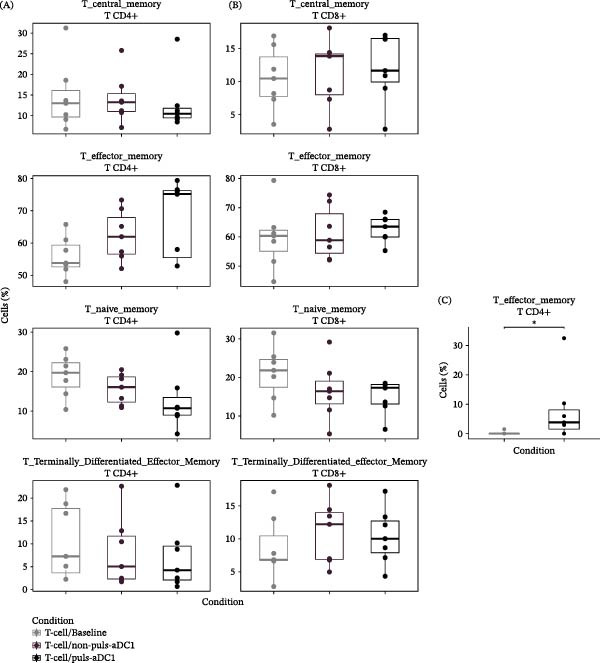
Phenotypic characterization of HIV‐specific T cells. T cell subsets and IFN‐γ production were analyzed by flow cytometry after coculture with αDC1. The graphs represent the percentage of naïve (CCR7+ CD45RA+) and memory T cell subpopulations: central memory (*T*_CM_) T cells (CCR7+ CD45RA−); effector memory (*T*_EM_) T cells (CCR7‐CD45RA‐); and terminally differentiated effector memory T cells (*T*_EMRA_) (CCR7‐CD45RA+). In (A) the percentage of CD4+ and (B) CD8^+^ T cells are represented; in (C), IFN‐γ production by CD4+ *T*_EM_ is shown. Autologous T cells maintained without stimulation (Baseline) (open circles); T cells sensitized by mature αDC1 (mat_αDC1; gray squares), or pulsed αDC1 (puls_αDC1) (black triangles). The horizontal lines indicate the medians of the studied conditions. Values were analyzed with the Mann–Whitney test. ( ^∗^ *p* < 0.05; *n* = 7).

Since 20‐day coculture allows access to the cellular memory profile, naïve T cells, and HIV‐specific memory T cells were studied next. The results obtained show a tendency toward a decrease in the naïve population when T lymphocytes sensitized by pulsed αDC1 are compared with autologous T cells maintained without stimulation, baseline T cells (19.7% CD4+ T baseline versus 10.7% CD4+ T sensitized by pulsed αDC1; 21.8% CD8+ T baseline versus 17.3% CD8+ T sensitized by pulsed αDC1); as well as, a trend toward an increase in the percentage of effector memory cells (TEM) for both, CD4+ and CD8+ T subpopulations (53.8% CD4+ T baseline versus 75.1% CD4+ T sensitized by pulsed αDC1; 60.4% CD8+ T baseline versus 63.5% CD8+ T sensitized by pulsed αDC1) were observed (Figure 5A,B). In fact, a negative correlation was found between naïve CD4+ T and CD4+ TEM from cocultures with pulsed αDC1 ( ^∗^ *p*  < 0.05) (Supporting Information 15: Figure S7). Moreover, CD4+ TEM sensitized by pulsed αDC1 were responsible for significantly higher production of IFN‐γ (3.8%) when compared with CD4^+^ T cells sensitized by mature αDC1 (0.01%) (*p*  < 0.05; Figure 5C).

## 4. Discussion

In the development of antiviral vaccines, the choice of antigens is pivotal to induce a specific immune response, capable of overcoming viral heterogeneity and promoting functional control of infection. In the present study, Gag and Pol peptide sequences used to pulse αDC1 were selected based on a previous study [29] that employed two complementary predictive tools: the graph‐based algorithm *Epigraph90* [36] and a structure‐based network analysis [37]. These approaches enabled the identification of highly conserved peptide regions with potential to induce CTL responses while simultaneously exerting topological constraints on the viral proteome that may compromise viral fitness. The epigraph approach identified 5 gag and 30 pol sequences, and network analysis selected 4 env, 2 nef, 12 pol, and 7 gag sequences. In addition to these, a complete set of HIV‐1 Gag peptides, composed of 34 sequences of conserved and nonconserved regions of Gag, was used as a reference response. Among these peptides, 7 out of 34 Gag complete set‐peptides were also identified by Network approach and three sequences of Pol were simultaneously identified by Epigraph and Network [29].

From these 84 HIV‐1 peptide pools tested by them, we selected the nine peptides (seven Gag and two Pol, Table 1) that elicited the strongest T cell responses in our cohort, as measured by IFN‐γ production. In our results using PBMCs, samples from all donors responded to at least three of these nine HIV‐1‐specific peptides.

It is important to note that we used all the same peptides tested in the Thai population, from which we selected the nine most effective ones, but unlike the cohort studied by Garcia‐Bates et al. [29], which included individuals who initiated treatment during early HIV‐1 infection (Fiebig stages I–IV), our Brazilian cohort was composed of individuals in the chronic phase of HIV‐1 infection. Despite this difference, we observed measurable HIV‐specific T cell responses. In fact, even among the 7 out of 23 participants who had a nadir lower than 250 cells/µL, PBMCs were able to respond to the peptides by producing IFN‐γ. Interestingly, CD8+ T from the two individuals (P04 and P11) with the lowest nadirs (25 and 7 cells/µL, respectively) (Table 2) were able to respond to at least eight of the nine peptides. Additionally, a higher frequency of CD4^+^ T cells responding to peptides was observed compared to CD8^+^ T cells (Supporting Information 16: Figure S8). Furthermore, as highly conserved regions were mapped to the tested sequences, we observed that our Brazilian cohort was also able to respond to the peptides selected for a Thai cohort, suggesting the coverage of a broad range of possible HLA‐associated haplotypes by these selected peptides.

Studies focusing on peptides for loading DCs published until now in general used conserved peptides or mRNA encoding HIV conserved peptides. On the other hand, selection of peptides based on ultraconserved sequences associated with the topological importance of these peptides constitutes an innovative approach with the potential to improve the antigen breadth. Our results suggested that these selected peptides can stimulate specific immune responses in a heterogeneous cohort as the Brazilian population.

The ability to control HIV‐1 replication in the absence of HAART is observed in a rare group of PLWH, called elite controllers (ECs) [38]. Several aspects have been proposed to explain this phenotype, including host genetic factors that restrict viral replication [39, 40]; innate immune responses, such as the actions of NK cells and DCs [41]; and adaptive immune responses marked by activity of CD8^+^ T cells, particularly polyfunctional CD8^+^ T cells with cytotoxic potential, have been studied as a functional cure model for HIV [42, 43]. It is known that the long‐term maintenance and quality of the HIV‐specific CD8+ memory T cell response depend on the support of CD4^+^ T cells, mediated in part by cytokines such as IFN‐γ and IL‐2 [38]. With regard to immune response induced by pulsed αDC1, our results showed a higher percentage of IFN‐γ production by CD4^+^ T cells compared to CD8^+^ T cells. Similarly, Garcia‐Bates et al. [29] observed higher HIV‐specific CD4^+^ T cell responses when 14–21mer peptide pools were used, while CD8^+^ T cell responses were better observed using 8–11mer HIV‐1 epitopes during coculture reestimulation. This is consistent with the known differences in MHC presentation: CD4^+^ T cells typically recognize longer peptides bound to MHC class II molecules of antigen‐presenting cells than those presented by MHC class I (8–10 aa), recognized by CD8^+^ T cells [44].

In therapeutic vaccine trials using DCs pulsed with HIV peptides have been observed several outcomes. Levy et al. [15] found polyfunctionality mainly in CD4^+^ T cells following immunization using lipopeptide‐pulsed DCs (Gag, Pol, and Nef), and this cellular response was associated with a decrease in viral load of vaccinated individuals. DCs pulsed with HIV peptides (Gag, Pol, Env, Vpu, and Vif) also induced viral‐specific CTL and CD4^+^ T cell responses in treatment‐naïve patients, with stronger CD4^+^ T cell responses compared with CD8+ T‐cell responses and a transient decrease in viral load [12]. In contrast, other studies observed HIV‐specific T cell responses in PLWH immunized with peptide‐pulsed DCs without virologic control during analytic treatment interruption (ATI) [18, 45]. Nevertheless, a clinical trial is currently ongoing to evaluate the safety and stimulatory potential of αDC1 pulsed with HIV peptides in comparison to αDC1 pulsed with inactivated virus [46]. Despite considerable differences in the protocols for obtaining DCs and the peptides used, most immunotherapies using peptide‐pulsed DCs alone or associated with adjuvants or immunomodulators found some degree of T cell immune response, not necessarily being associated with the control of viral proliferation rebound.

Another important point to be highlighted is the choice of methodology used to verify T cell responses. Although 6 days of coculture with αDC1 were not able to stimulate autologous responses, on the other hand, increased production of IFN‐γ was observed after 20 days, with the subpopulation of effector memory CD4^+^ T cells (TEM) being responsible for the production of IFN‐γ after reexposure to the HIV antigens. Extending the time for culture proved to be an important experimental lever in revealing the population of memory cells, which are essential for promoting long‐term protection and controlling and eradicating foreign pathogens. Sensitization of immune cells by DCs followed by restimulation with specific peptides is capable of rapidly expanding effector memory CD4 T cells. In fact, these cells were demonstrated to be able to respond immediately upon reinfection, migrating to the inflamed peripheral tissues or B‐cell areas [47, 48]. On the other hand, we were not able to demonstrate the induction of CD8 T cell response mediated by shorter peptides, as demonstrated by Garcia‐Bates et al. [29] Since we observed an increased response in two out of three samples, this fact may be attributed to the small sample size, which impaired the interpretation of our test response.

We acknowledge there are several limitations of this study. Peptide selection was performed using samples from 11 study participants; however, validation of the response through IFN‐gamma or GzB production by T lymphocytes stimulated by pulsed αDC1 with the previously selected peptides was performed with samples from only three to seven individuals. Although we were able to demonstrate statistical significance regarding IFN‐gamma production by CD4 T lymphocytes in response to long peptides, increased sample size is required to generalize to the larger population. Also, it remains to be determined whether stimulation with shorter peptides could be able to generate TCD8 response.

In this context, although Figures 3 and 4 demonstrated detectable responses in approximately half of the individuals included in the study, this finding is consistent with recent reports in people living with HIV under effective ART. Longitudinal and functional studies have shown that HIV‐specific T cell responses can be detected and remain stable overtime, even in small cohorts of individuals with prolonged viral suppression, indicating that responses of moderate magnitude can be biologically relevant and informative in immunotherapy studies. Moreover, analyses of T cell responses in individuals receiving ART indicate that, although the magnitude and breadth of these responses are often reduced compared to untreated individuals, they remain detectable and directed against multiple HIV epitopes, preserving immunological relevance. The detection of anti‐HIV cellular responses exhibits interindividual variability, even when highly immunogenic epitopes and sensitive methodologies are employed, reflecting differences in HLA repertoire, history of viral exposure, and immune status [49, 50]. In this context, studies based on ELISPOT and functional flow cytometry have reported measurable responses in ~40%–70% of participants, without compromising biological interpretation [51]. Thus, although not all individuals of our cohort responded to each peptide individually, response frequencies around 50% are consistent with the current literature and can reflect immunological relevance.

Overall, our results are suggestive of the potential of αDC1 to stimulate a T cell immune response in vitro. It remains to be determined whether this stimulation could be sufficient to effectively act on viral load or even latency. Additional investigations with a larger number of samples are needed to confirm whether activated CD8^+^ T cells can inhibit viral replication or whether the use of samples from chronically infected PLWH has depleted or dysfunctional CTLs. For that, the next step is to generate specific CD8 T cells through initial stimulation with larger peptides presented by αDC1 and restimulation with shorter peptides. The potential of these CD8 T cells to inhibit viral replication could be done through coculture of these cells with infected CD4 T cells, analyzing the capacity to promote cell lysis. Confirmation of the effectiveness of CD8 T lymphocytes restimulated by short peptides in inhibiting viral replication would suggest the capacity of the immune response to control viral load.

Furthermore, methodological modifications to the vaccine product, such as incorporation of HIV‐derived epitopes alongside Gag and Pol peptides during the αDC1 pulsing, may enhance the induction of CD8^+^ T cell responses in addition to CD4^+^ T cell activation. Since the present study precedes a clinical trial to evaluate DC‐based immunotherapy for the treatment of chronically HIV‐infected individuals, defining suitable peptides for DC loading is a fundamental step in establishing an appropriate protocol.

## 5. Conclusions

Our results are suggestive of the potential of αDC1, pulsed with the optimized peptides, to stimulate a T cell immune response in vitro in a Brazilian cohort. This knowledge is important considering a future clinical trial to evaluate the ability of these αDC1 to stimulate an effective immune response against HIV.

It remains to be determined whether CD8 T cells will be able to inhibit viral replication. Such adjustments could be fundamental to achieving the goal of developing an effective immunotherapy based on DCs.

## Author Contributions

Laís Teodoro Da Silva responsible for the conceptualization of the study, performed the experimental assays and interpretation of experimental results and wrote the original manuscript draft. Marina Mazzilli Ortega contributed to the conceptualization of the study, performed the experimental assays, and participated in writing the manuscript. Silvia de Jesus Mota performed experimental assays. Guilherme Castellani contributed to the execution of experimental assays. Dennyson Leandro M. Fonseca performed formal data analysis and interpretation of the experimental results. Larissa Danielle Bahls Pinto conducted HLA typing of patient samples. Gabriela Justamante Handel Schmitz contributed to the execution of experimental assays. Isabela Leite contributed to data curation and provided biological samples for HLA genotyping. Theo Leite contributed to data curation and provided biological samples for HLA genotyping. Mariana Amelia Monteiro was responsible for patient recruitment and sample collection. Sandra Marcia Muxel performed formal data analysis. Robbie B. Mailliard contributed to the conceptualization and methodological framework of the study, participated in the writing and critical revision of the manuscript, provided scientific guidance. Alberto José da Silva Duarte provided overall scientific guidance. Telma Miyuki Oshiro led the conceptualization of the study, supervised all stages of the project, contributed to manuscript writing, provided scientific guidance, and was responsible for project administration.

## Funding

This work was supported by the Sao Paulo Foundation Research, FAPESP (Grants 16/25212‐9 and 24/14088‐1). Laís Teodoro Da Silva is the recipient of a fellowship from FAPESP (Grant 23/11990‐3). Marina Mazzilli Ortega is the recipient of a fellowship from FAPESP (Grant 23/14374‐1). Silvia de Jesus Mota is the recipient of a fellowship from CAPES (Grant 88887.970045/2024‐00) and FAPESP (Grant 25/12297‐5). Guilherme Castellani is the recipient of a fellowship from CAPES (Grant 88887.196649/2025‐00) and FAPESP (Grant 25/13425‐7). Dennyson Leandro M. Fonseca is the recipient of a fellowship from FAPESP (Grant 25/03433‐2). Larissa Danielle Bahls Pinto is the recipient of a fellowship from CSD‐UEM (Grant 1589/2017). Gabriela Justamante Handel Schmitz is the recipient of a fellowship from FAPESP (Grant 21/06139‐7). Isabela Leite is the recipient of a fellowship from CAPES (Grant 138377/2025‐1). Theo Leite is the recipient of a fellowship from CAPES (Grant 88887.251198/2026‐00). Sandra Marcia Muxel is the recipient of a fellowship from FAPESP (Grants 24/08802‐3 and 24/08360‐0) and from the National Council for Scientific and Technological Development‐CNPq (Grant 303384/2024‐7). Robbie B. Mailliard is supported by the NIH/NAID (Grants R01 AI152655) and the Case/UHC‐Pitt Center for AIDS Research (Rustbelt CFAR) (Grant 2P30AI036219‐26A1). Telma Miyuki Oshiro and Alberto José da Silva Duarte are recipients of a fellowship from the National Council for Scientific and Technological Development‐CNPq (Grants 304043/2022‐2 and 305135/20198, respectively).

## Disclosure

A previous version of this manuscript has been made available as a preprint at Wiley Open Research (doi 10.22541/au.175697120.07287564/v1).

## Conflicts of Interest

The authors declare no conflicts of interest.

## Supporting Information

Additional supporting information can be found online in the Supporting Information section.

## Supporting information


**Supporting Information 1** Figure S1 Characterization of αDC1 immunophenotypic and IL12‐p70 production. (A) Immature DCs (iDCs) and αDC1 were labeled with monoclonal antibodies against CD40, CD83, HLA‐DR, CCR7, and CD86. Bar graphs show the median and interquartile range of the percentage of viable CD14− CD11c+ cells (DCs). (B) On Days 5 and 7 of culture, iDCs and αDC1 were harvested and incubated for 24 h with rCD40L, after which supernatants were collected for IL‐12p70 quantification by ELISA. The graph shows IL‐12p70 concentrations (pg/mL) produced by iDCs (white circles) and αDC1 (black circles), with horizontal lines indicating medians. Values were analyzed using the Mann–Whitney test ( ^∗∗^ *p*  < 0.005; *n* = 8).

**Supporting Information 2** Figure S2 Phenotypic and viability comparison of monocyte‐derived dendritic cells generated by two different methods. Bar graphs represent the frequency (median; IQR 75th and 25th) of viable cells (A) and CD14‐CD11c+ cells (B), as well as the expression of CD40 and HLA‐DR (C) in immature DCs (iDC) and mature DCs (mDC) generated from monocytes obtained by adherence or bead isolation (n = 3). Light gray bars represent iDC generated by adherence; black bars represent mDC generated by adherence; dotted light gray bars represent iDC generated by bead isolation; dotted dark gray bars represent mDC generated by bead isolation. Comparisons between two groups were performed using the Wilcoxon matched‐pairs signed‐rank test. For multiple comparisons, Friedman’s test followed by Dunn’s post‐test was applied.

**Supporting Information 3** Table S1 Pools of HIV‐1 epitopes for the Gag and Pol regions.

**Supporting Information 4** Figure S3 Representative strategy to analyze IFN‐γ production by T cells. Initially, PE x time was used to exclude any electronic noise during sample acquisition (A). In (B) a gate was applied to eliminate double cells from the analysis; then, the size (SSC) and granularity (FSC) patterns of the cells were analyzed (C); after that, within the live cell population (D), CD3+ populations were designed to define T lymphocytes (E) and, within this population, CD4+ and CD8+ T lymphocytes were selected (F). Finally, IFN‐γ production baseline (unstimulated T cells) (G), and IFN‐γ production by T cells cocultured with αDC1 pulsed with HIV‐1 peptides (H) are shown. The production of IFN‐γ by the CD4+ T subpopulation is represented; however, the same analysis was performed for CD8+ T lymphocytes. Sample acquisition was performed on an LSR Fortessa (BD) flow cytometer and data were analyzed using FlowJo v. 10 software.

**Supporting Information 5** Table S2 Effect size and normality test of HIV‐specific T cell response induced by αDC1 data.

**Supporting Information 6** Table S3 Effect size and normality test of CTLs induced by αDC1 data.

**Supporting Information 7** Table S4 Effect size and normality test of CD4+ and CD8^+^ T cell memory subsets after αDC1 stimulation.

**Supporting Information 8** Table S5 Significance analysis of HIV‐specific T cell response induced by αDC1 data.

**Supporting Information 9** Table S6 Significance analysis of CTLs induced by αDC1 data.

**Supporting Information 10** Table S7 Significance analysis of phenotypic characterization of HIV‐specific T‐cells data.

**Supporting Information 11** Figure S4 Effect size and distribution analysis of producing CD4+ and CD8^+^ T cells after αDC1 stimulation. (A) Forest plots showing the effect size (Wilcoxon *r*) of IFN‐y production by CD4+ and CD8^+^ T cells after 6 and 20 days (B) of coculture with αDC1. Each point represents the effect size calculated, horizontal bars indicate confidence intervals. (C–D) Quantile–quantile (Q–Q) plots illustrate the distribution of IFN‐y producing CD4+ and CD8^+^ T cells at Day 6 (C) and Day 20 (D). (E) Q–Q plots of Granzyme‐B expressing CD4+ and CD8^+^ T cells at Day 20. Gray symbols represent CD4^+^ T cells and black symbols represent CD8^+^ T cells. Shaded areas indicate the 95% confidence interval of the fitted regression line.

**Supporting Information 12** Figure S5 Effect size and distribution analysis of CD4+ and CD8^+^ T cell memory subsets after αDC1 stimulation. (A) Forest plots showing the effect size (Wilcoxon *r*) for the frequency of CD4+ and CD8^+^ T cell memory subsets after αDC1 stimulation, including naive, central, effector, and terminally differentiated effector memory. Comparisons were made between DC after vs. baseline, mDC vs. baseline, and pulsed vs. mat_αDC1. Each point represents the estimated effect size, and horizontal bars indicate confidence intervals. Gray symbols represent CD4^+^ T cells, and black symbols represent CD8^+^ T cells. (B) Quantile–quantile (Q–Q) plots illustrating the distribution of CD4+ and CD8^+^ T cell memory subsets. Shaded areas indicate the 95% confidence interval of the fitted regression line.

**Supporting Information 13** Figure S6 Distribution analysis of CD4+ and CD8^+^ T cell responses across stimulation conditions. Quantile‐Quantile (Q–Q) plots showing the distribution of CD4+ and CD8^+^ T cell frequencies under different conditions: baseline, DC_afer, DC_efer and mDC. The upper panels represent CD4^+^ T cells, and the lower panels represent CD8^+^ T cells. Shaded areas indicate the 95% confidence interval of the fitted regression line.

**Supporting Information 14** Table S8 IFN‐γ CD4+ and CD8^+^ T cells production per donor (log scale).

**Supporting Information 15** Figure S7 Correlation analysis. Negative correlation between naïve CD4+ T and CD4+ TEM from cocultures with pulsed αDC1 ( ^∗^ *p*  < 0.05) is shown. The correlation was established using Spearman’s correlation (*r* ^2^ > 0.7 and *p*  < 0.05; *n* = 7).

**Supporting Information 16** Figure S8 Comparison of the frequency of HIV‐specific CD4+ and CD8^+^ T cell responses. PBMCs individually stimulated with 1 µg/mL 14‐21mer HIV‐1 peptide for 6 days were analyzed for detection of IFN‐γ production. The frequency of positive peptide responses is represented. Each symbol represents a peptide tested. The horizontal lines indicate the medians of the studied conditions. Values were analyzed with the Wilcoxon matched‐pairs signed‐rank test. ( ^∗^ *p* = 0.039; *n* = 11).

## Data Availability

The data that support the findings of this study are available in the Supporting Information of this article.
