# Supplementary figures and images for: Induction of HIV‐Specific T Cell Responses Using αDC1 Pulsed With Conserved HIV‐1 Peptides
Source: J Immunol Res. 2026 May 15;2026:5457670. doi: 10.1155/jimr/5457670 (PMC13177841; doi:10.1155/jimr/5457670)

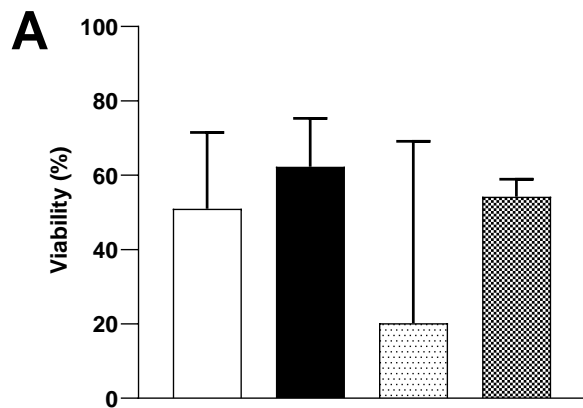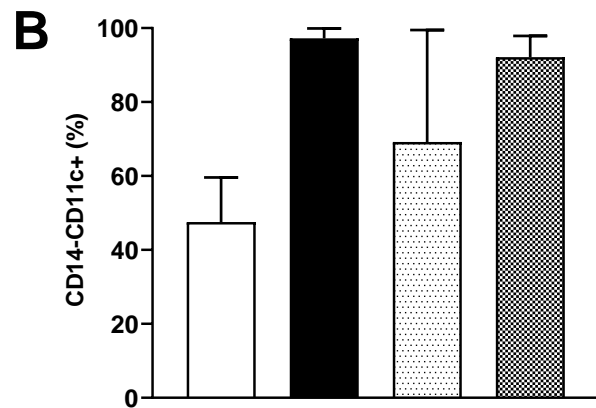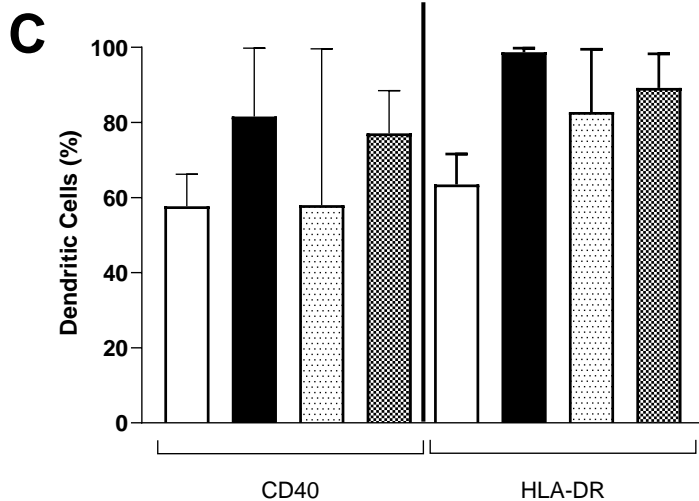

□ iDC adherence  
■ mDC adherence  
▤ iDC bead  
▥ mDC bead

Supplement: Supplementary file 2 — Supporting Information 2 Figure S2 Phenotypic and viability comparison of monocyte‐derived dendritic cells generated by two different methods. Bar graphs represent the frequency (median; IQR 75th and 25th) of viable cells (A) and CD14‐CD11c+ cells (B), as well as the expression of CD40 and HLA‐DR (C) in immature DCs (iDC) and mature DCs (mDC) generated from monocytes obtained by adherence or bead isolation (n = 3). Light gray bars represent iDC generated by adherence; black bars represent mDC generated by adherence; dotted light gray bars represent iDC generated by bead isolation; dotted dark gray bars represent mDC generated by bead isolation. Comparisons between two groups were performed using the Wilcoxon matched‐pairs signed‐rank test. For multiple comparisons, Friedman’s test followed by Dunn’s post‐test was applied. [file JIMR-2026-5457670-s006.pdf]

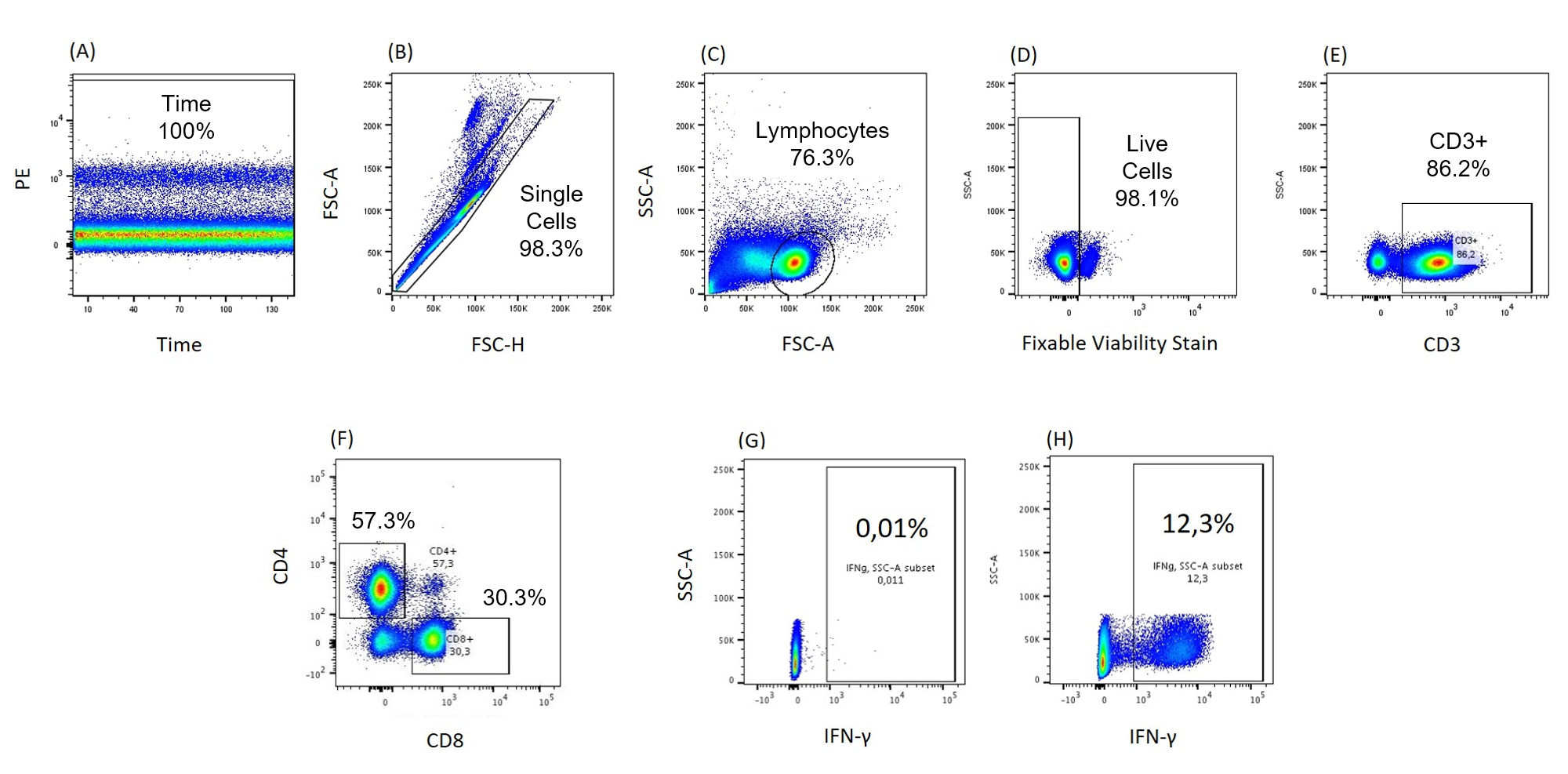

Supplement: Supplementary file 4 — Supporting Information 4 Figure S3 Representative strategy to analyze IFN‐γ production by T cells. Initially, PE x time was used to exclude any electronic noise during sample acquisition (A). In (B) a gate was applied to eliminate double cells from the analysis; then, the size (SSC) and granularity (FSC) patterns of the cells were analyzed (C); after that, within the live cell population (D), CD3+ populations were designed to define T lymphocytes (E) and, within this population, CD4+ and CD8+ T lymphocytes were selected (F). Finally, IFN‐γ production baseline (unstimulated T cells) (G), and IFN‐γ production by T cells cocultured with αDC1 pulsed with HIV‐1 peptides (H) are shown. The production of IFN‐γ by the CD4+ T subpopulation is represented; however, the same analysis was performed for CD8+ T lymphocytes. Sample acquisition was performed on an LSR Fortessa (BD) flow cytometer and data were analyzed using FlowJo v. 10 software. [file JIMR-2026-5457670-s008.docx]

**A**

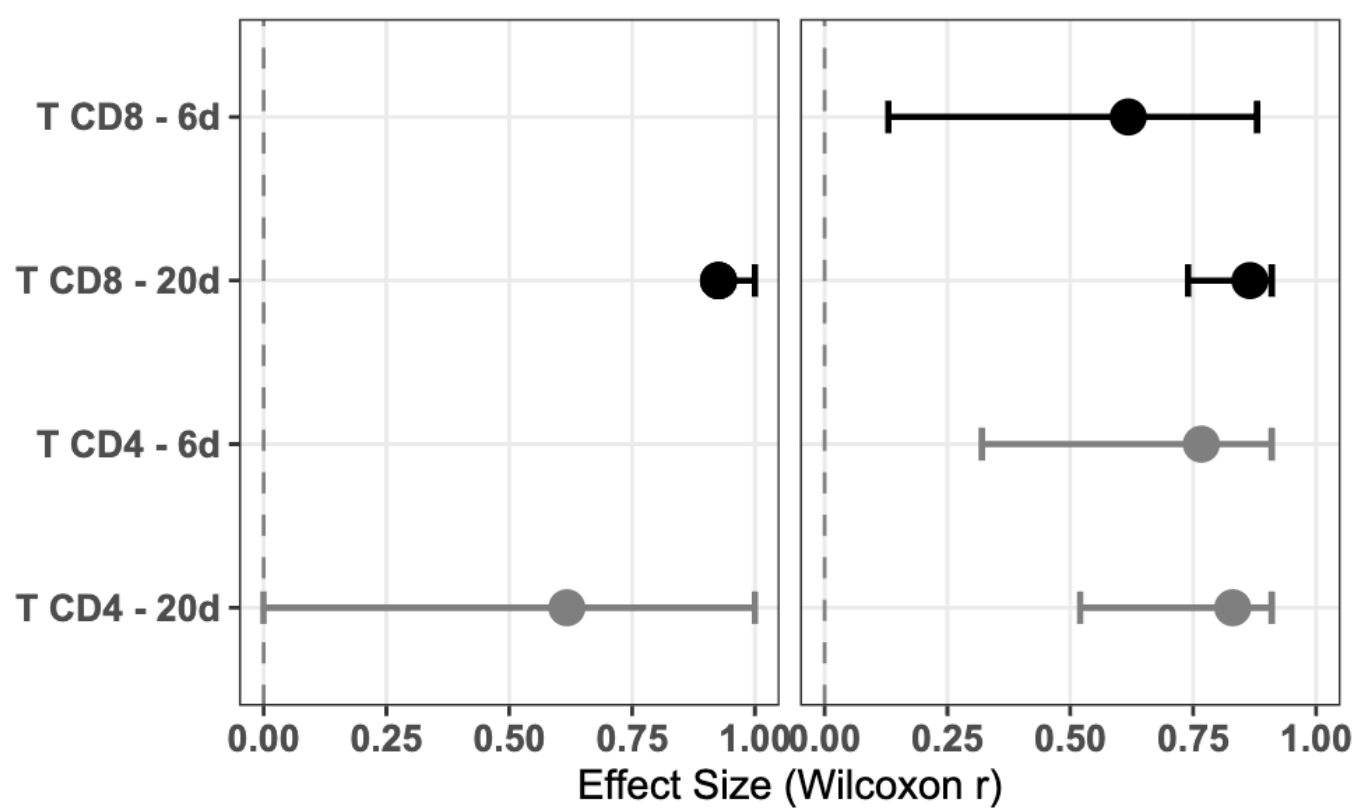

Cell Type ● T CD4 ● T CD8

**B**

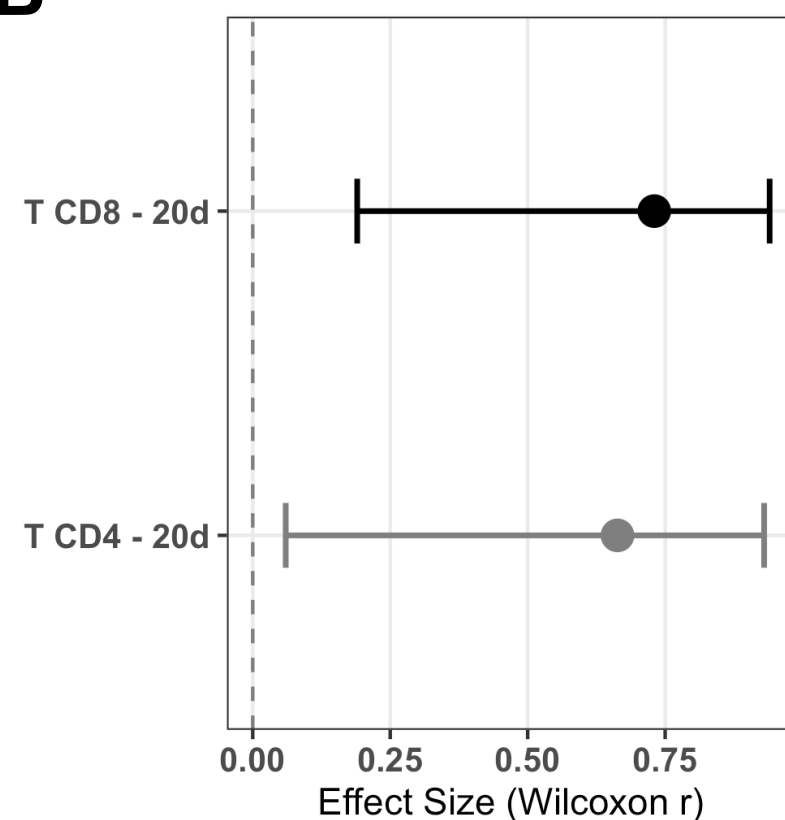

Cell Type ● T CD4 ● T CD8

**C**

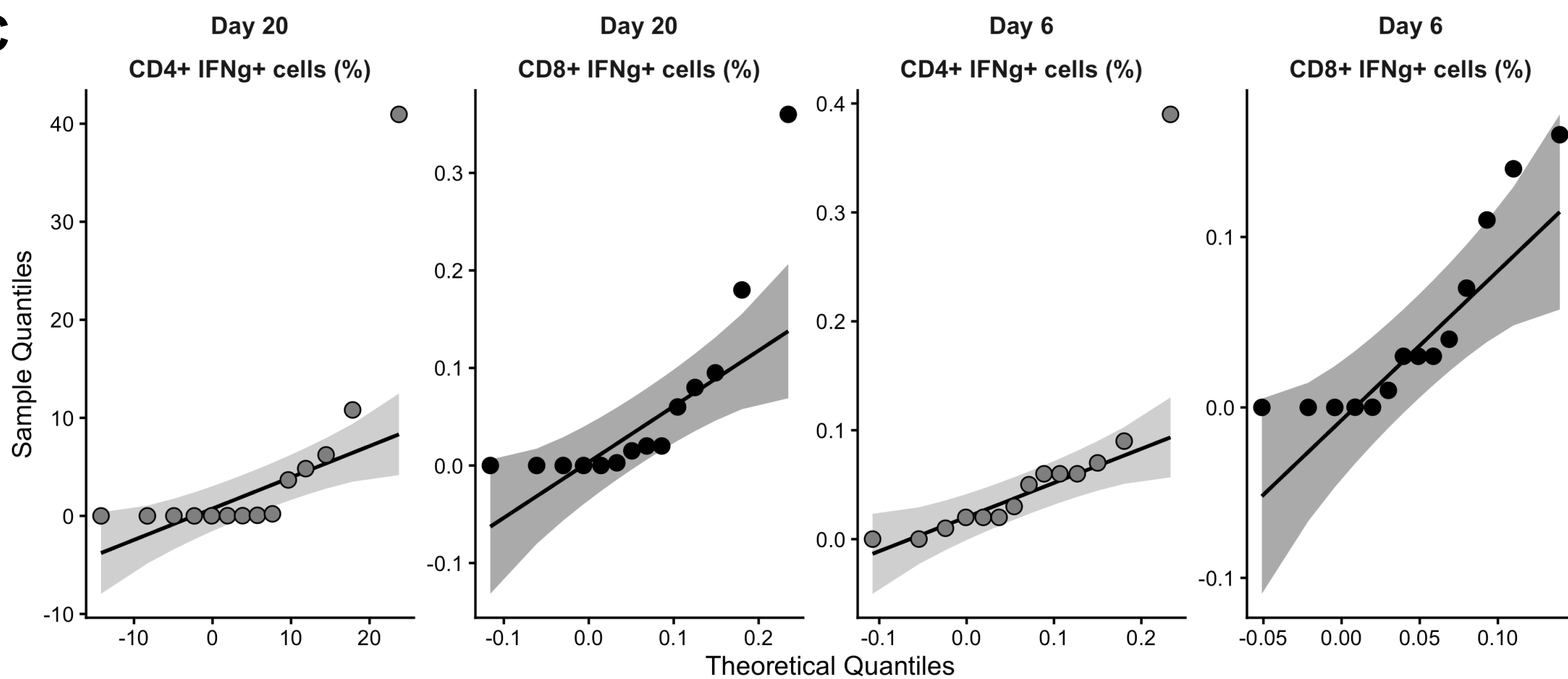

cell\_type ● T CD4 ● T CD8

**D**

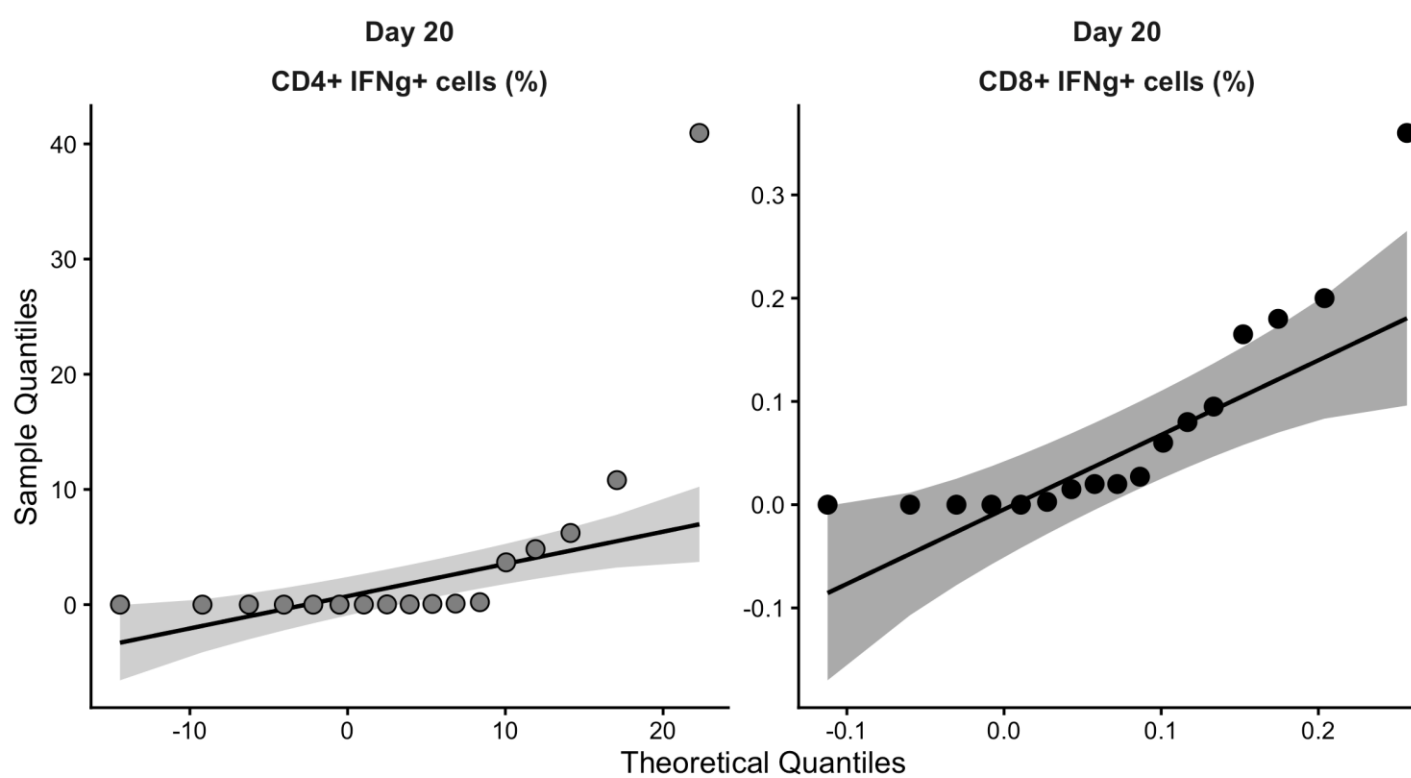

cell\_type ● T CD4 ● T CD8

**E**

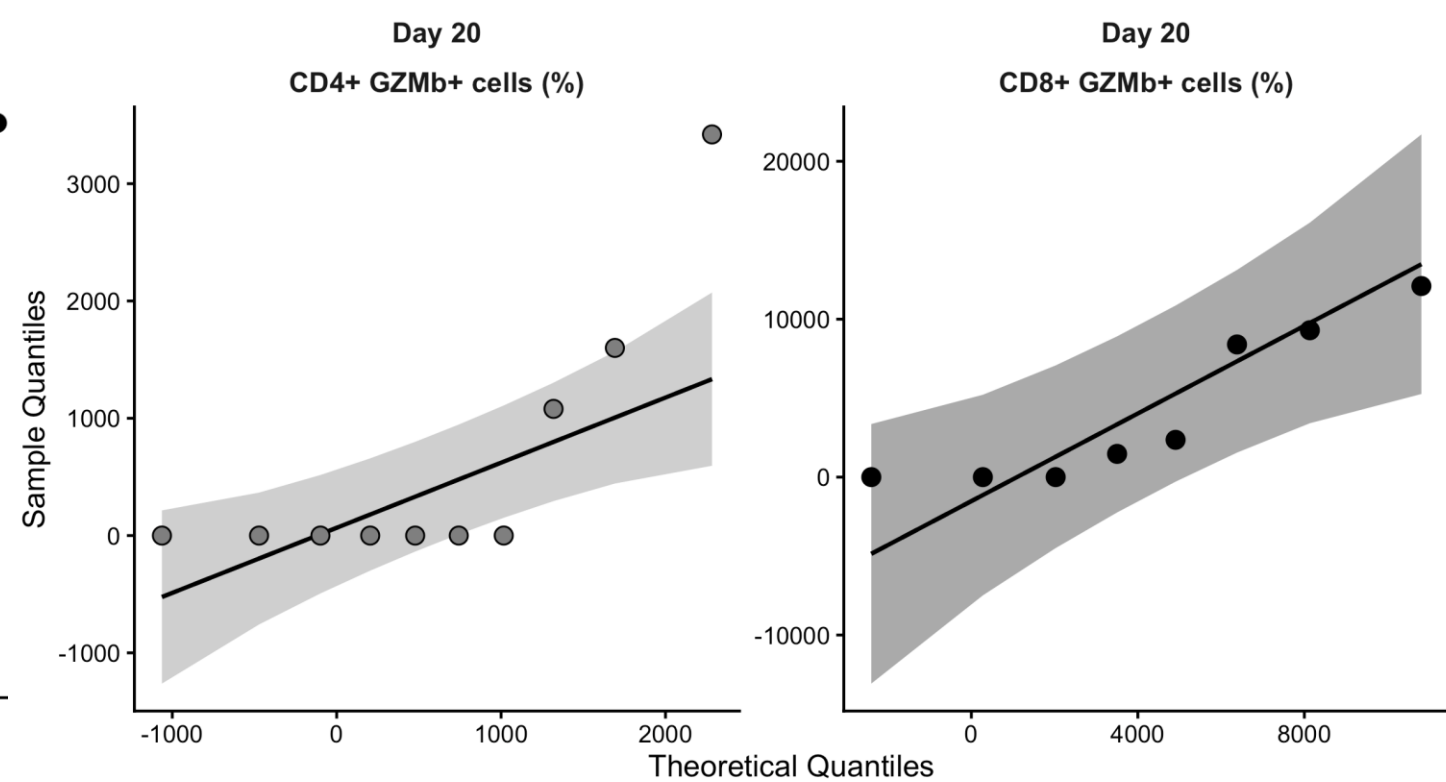

cell\_type ● T CD4 ● T CD8

Supplement: Supplementary file 11 — Supporting Information 11 Figure S4 Effect size and distribution analysis of producing CD4+ and CD8+ T cells after αDC1 stimulation. (A) Forest plots showing the effect size (Wilcoxon r) of IFN‐y production by CD4+ and CD8+ T cells after 6 and 20 days (B) of coculture with αDC1. Each point represents the effect size calculated, horizontal bars indicate confidence intervals. (C–D) Quantile–quantile (Q–Q) plots illustrate the distribution of IFN‐y producing CD4+ and CD8+ T cells at Day 6 (C) and Day 20 (D). (E) Q–Q plots of Granzyme‐B expressing CD4+ and CD8+ T cells at Day 20. Gray symbols represent CD4+ T cells and black symbols represent CD8+ T cells. Shaded areas indicate the 95% confidence interval of the fitted regression line. [file JIMR-2026-5457670-s016.pdf]

**A**

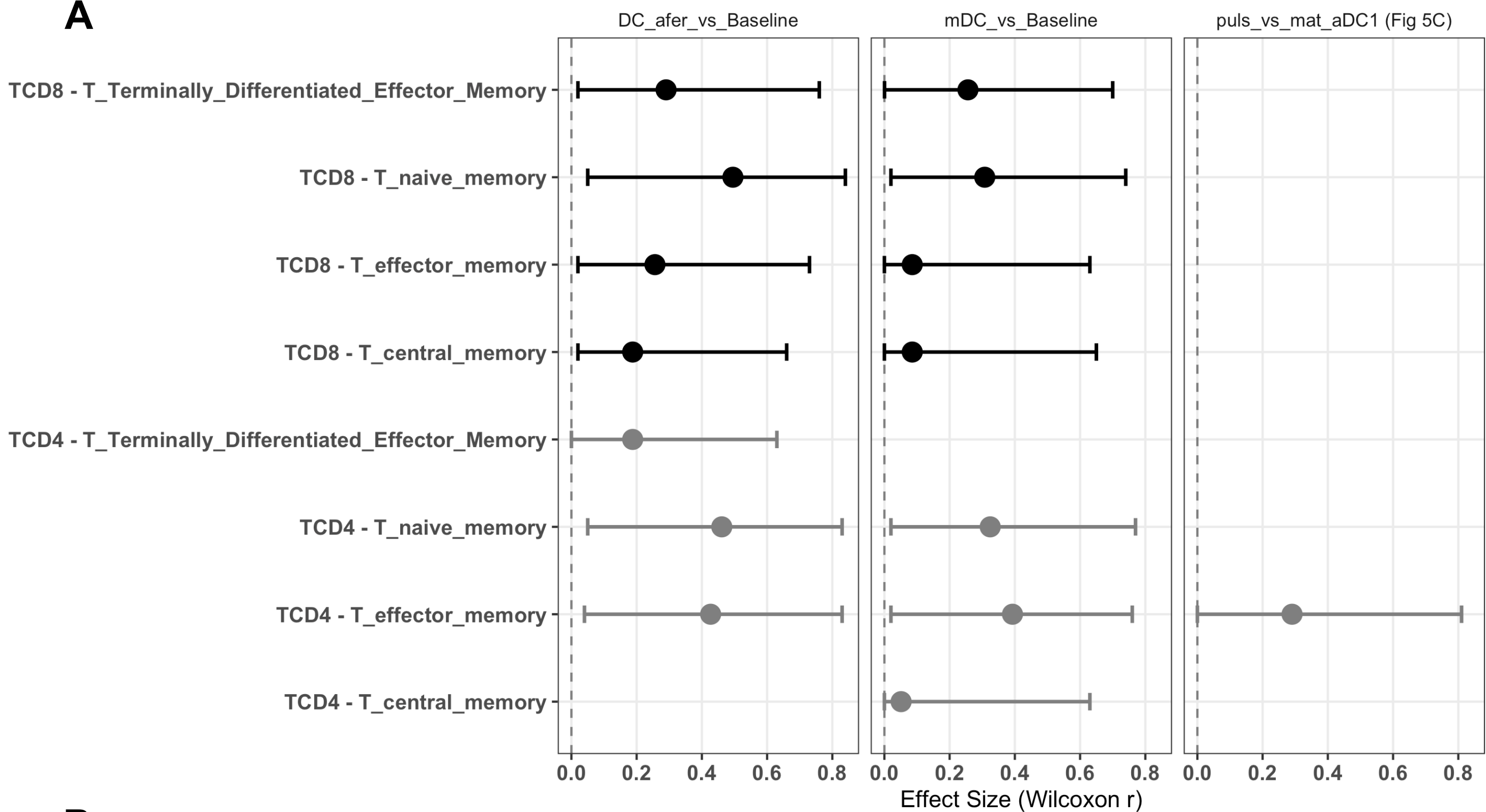

**B**

Cell Type ● TCD4 ● TCD8

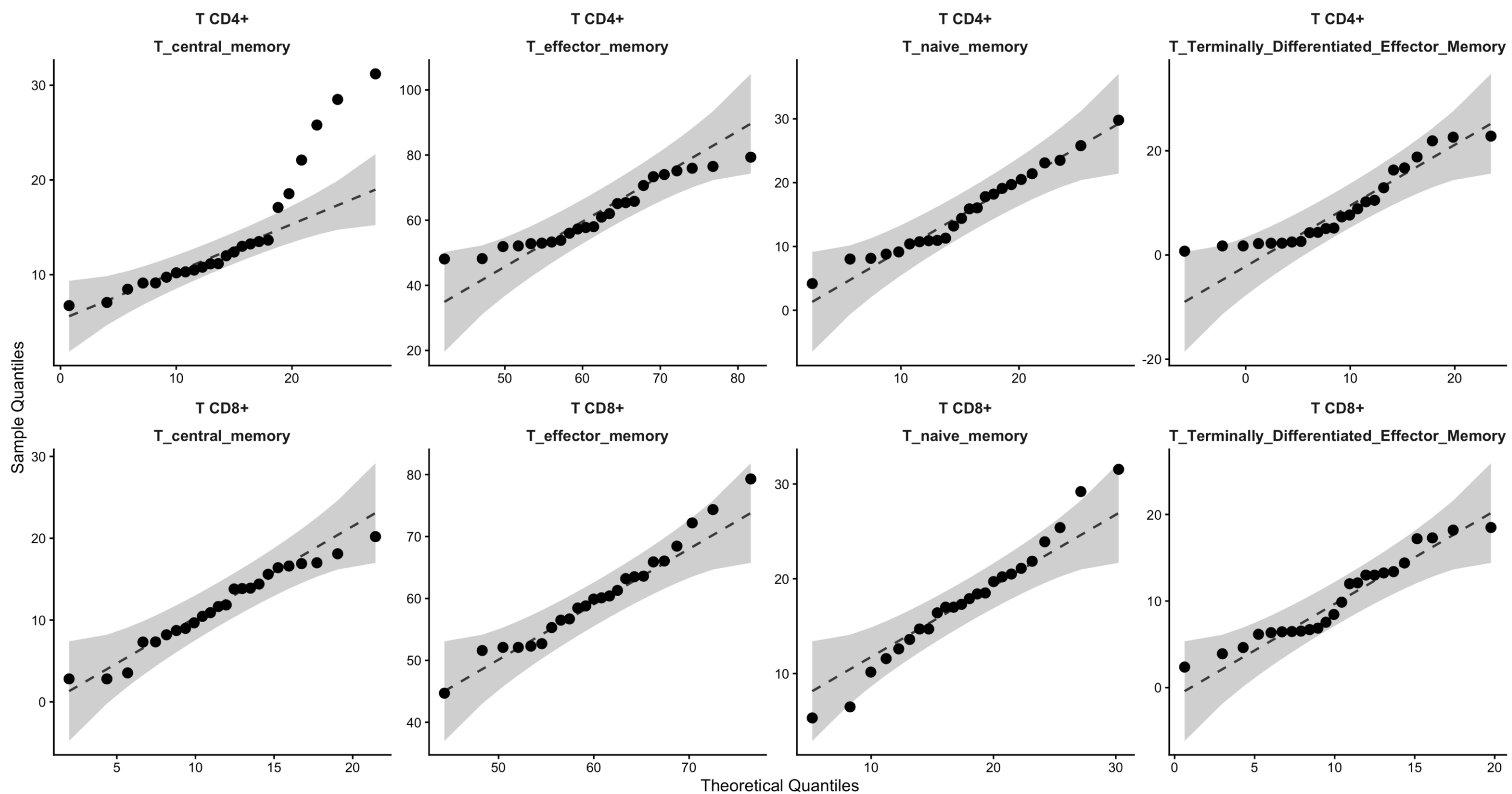

Supplement: Supplementary file 12 — Supporting Information 12 Figure S5 Effect size and distribution analysis of CD4+ and CD8+ T cell memory subsets after αDC1 stimulation. (A) Forest plots showing the effect size (Wilcoxon r) for the frequency of CD4+ and CD8+ T cell memory subsets after αDC1 stimulation, including naive, central, effector, and terminally differentiated effector memory. Comparisons were made between DC after vs. baseline, mDC vs. baseline, and pulsed vs. mat_αDC1. Each point represents the estimated effect size, and horizontal bars indicate confidence intervals. Gray symbols represent CD4+ T cells, and black symbols represent CD8+ T cells. (B) Quantile–quantile (Q–Q) plots illustrating the distribution of CD4+ and CD8+ T cell memory subsets. Shaded areas indicate the 95% confidence interval of the fitted regression line. [file JIMR-2026-5457670-s013.pdf]

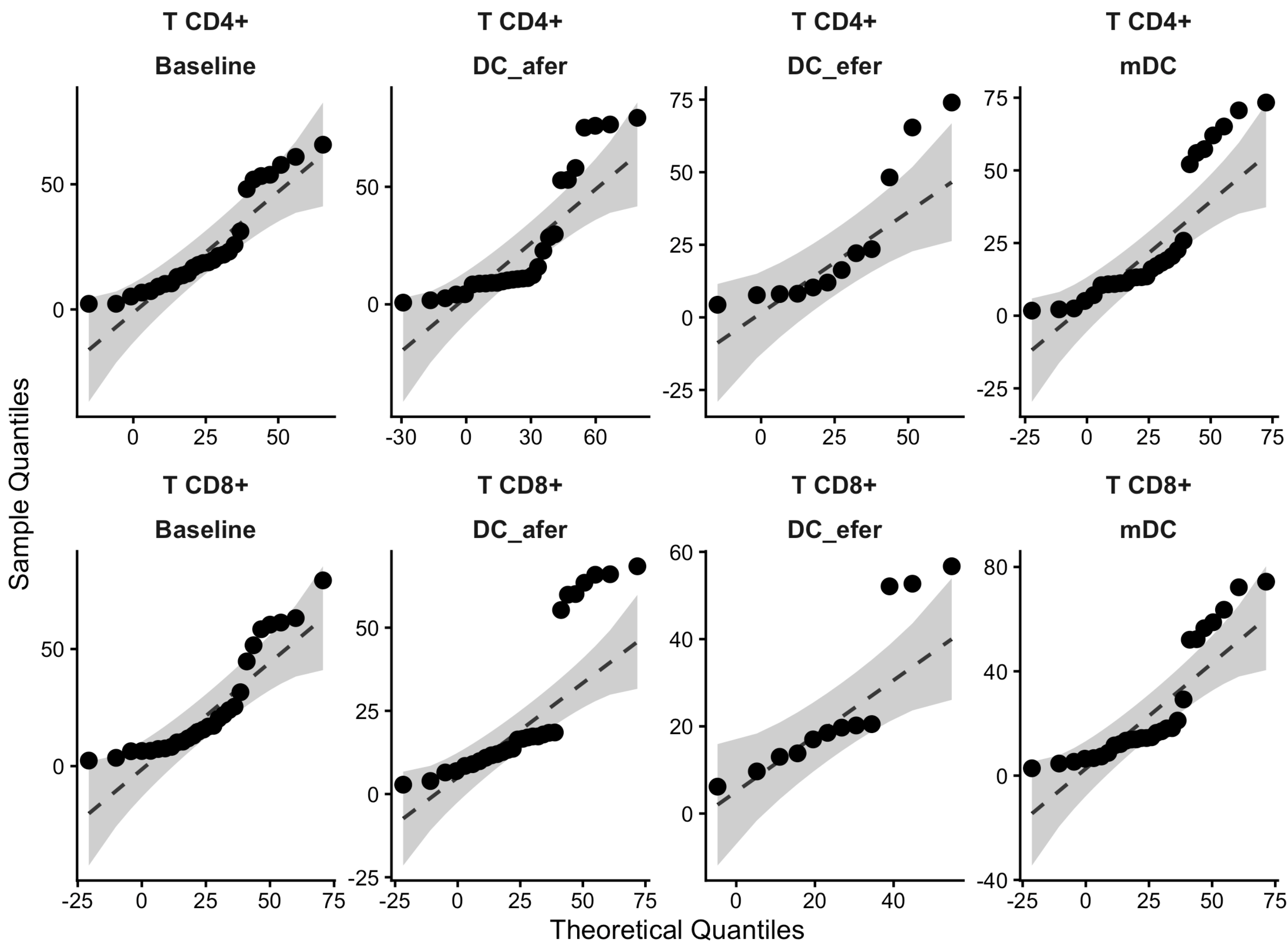

Supplement: Supplementary file 13 — Supporting Information 13 Figure S6 Distribution analysis of CD4+ and CD8+ T cell responses across stimulation conditions. Quantile‐Quantile (Q–Q) plots showing the distribution of CD4+ and CD8+ T cell frequencies under different conditions: baseline, DC_afer, DC_efer and mDC. The upper panels represent CD4+ T cells, and the lower panels represent CD8+ T cells. Shaded areas indicate the 95% confidence interval of the fitted regression line. [file JIMR-2026-5457670-s012.pdf]

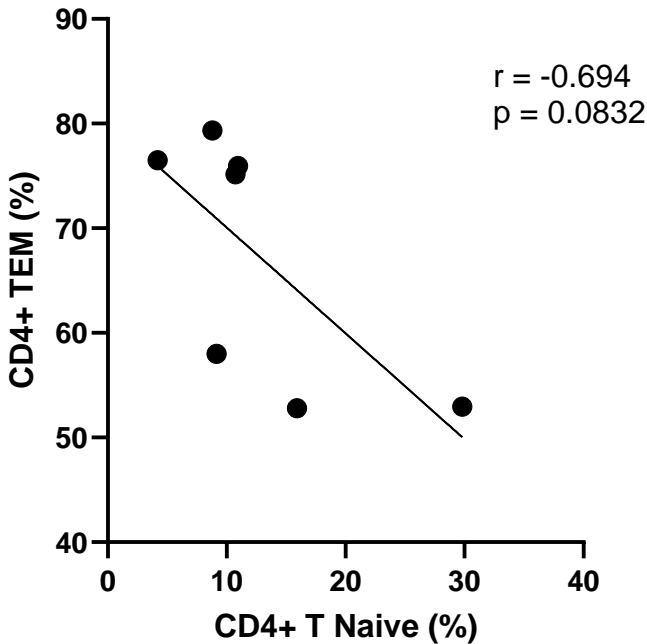

Supplement: Supplementary file 15 — Supporting Information 15 Figure S7 Correlation analysis. Negative correlation between naïve CD4+ T and CD4+ TEM from cocultures with pulsed αDC1 ( ∗ p < 0.05) is shown. The correlation was established using Spearman’s correlation (r 2 > 0.7 and p < 0.05; n = 7). [file JIMR-2026-5457670-s010.pdf]

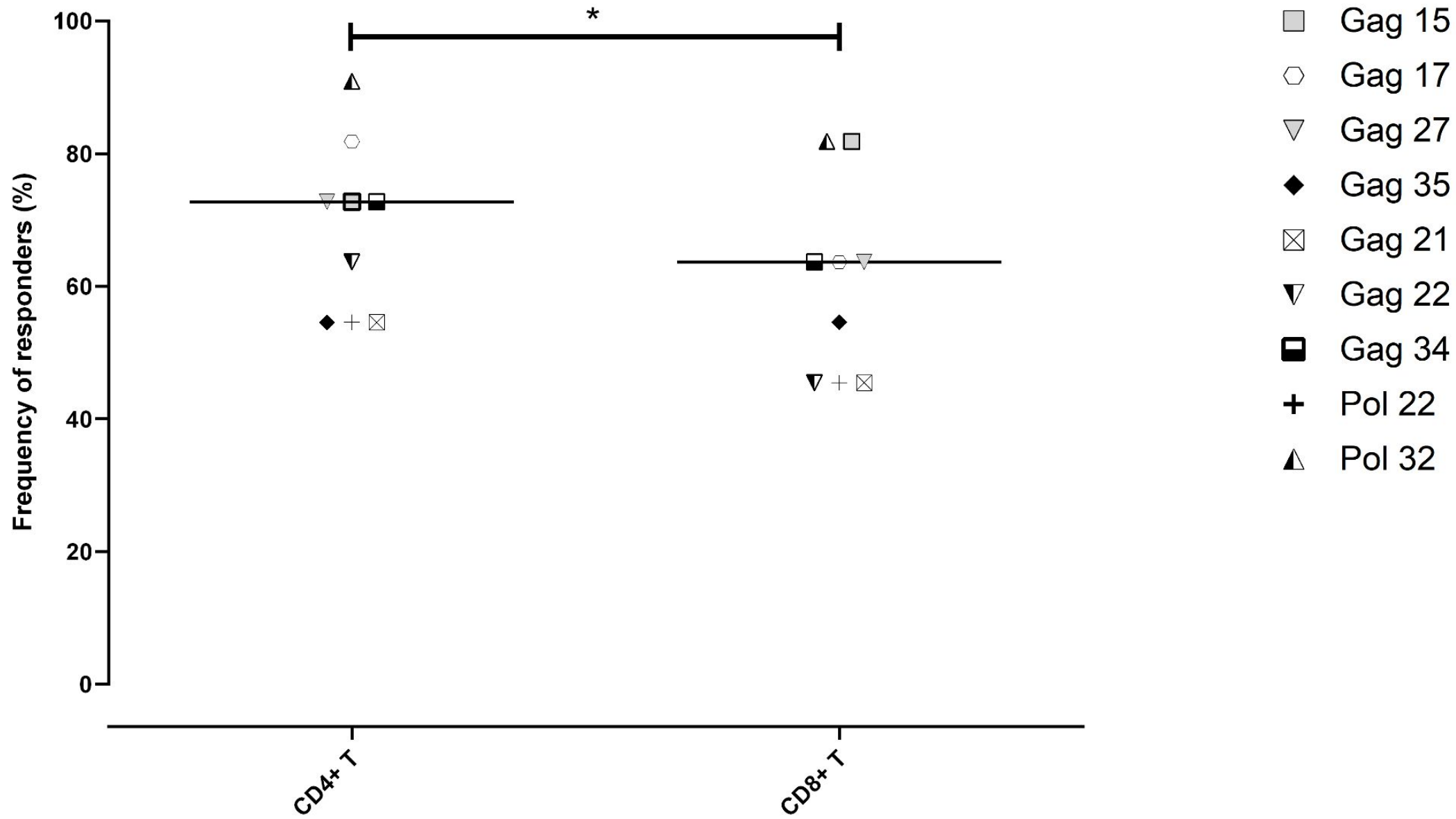

Supplement: Supplementary file 16 — Supporting Information 16 Figure S8 Comparison of the frequency of HIV‐specific CD4+ and CD8+ T cell responses. PBMCs individually stimulated with 1 µg/mL 14‐21mer HIV‐1 peptide for 6 days were analyzed for detection of IFN‐γ production. The frequency of positive peptide responses is represented. Each symbol represents a peptide tested. The horizontal lines indicate the medians of the studied conditions. Values were analyzed with the Wilcoxon matched‐pairs signed‐rank test. ( ∗ p = 0.039; n = 11). [file JIMR-2026-5457670-s009.pdf]
