# Supplementary material for: Induction of HIV‐Specific T Cell Responses Using αDC1 Pulsed With Conserved HIV‐1 Peptides
Source: J Immunol Res. 2026 May 15;2026:5457670. doi: 10.1155/jimr/5457670 (PMC13177841; doi:10.1155/jimr/5457670)
Supplement: Supplementary file 3 — Supporting Information 3 Table S1 Pools of HIV‐1 epitopes for the Gag and Pol regions. [file JIMR-2026-5457670-s007.docx]

| **Epitopes** | **Sequences** |
| --- | --- |
| G15c | EVIPMFSAL |
| G17d | ATPQDLNMML |
| G27a | FRDYVDRFY |
| G34a | IMMQRGNFK |
| P22b | HVASGYIEA |
| P32b | NNETPGIRYQY |
| P32c | IRYQYNVL |
