# Supplementary material for: Induction of HIV‐Specific T Cell Responses Using αDC1 Pulsed With Conserved HIV‐1 Peptides
Source: J Immunol Res. 2026 May 15;2026:5457670. doi: 10.1155/jimr/5457670 (PMC13177841; doi:10.1155/jimr/5457670)
Supplement: Supplementary file 1 — Supporting Information 1 Figure S1 Characterization of αDC1 immunophenotypic and IL12‐p70 production. (A) Immature DCs (iDCs) and αDC1 were labeled with monoclonal antibodies against CD40, CD83, HLA‐DR, CCR7, and CD86. Bar graphs show the median and interquartile range of the percentage of viable CD14− CD11c+ cells (DCs). (B) On Days 5 and 7 of culture, iDCs and αDC1 were harvested and incubated for 24 h with rCD40L, after which supernatants were collected for IL‐12p70 quantification by ELISA. The graph shows IL‐12p70 concentrations (pg/mL) produced by iDCs (white circles) and αDC1 (black circles), with horizontal lines indicating medians. Values were analyzed using the Mann–Whitney test ( ∗∗ p < 0.005; n = 8). [file JIMR-2026-5457670-s005.pdf]

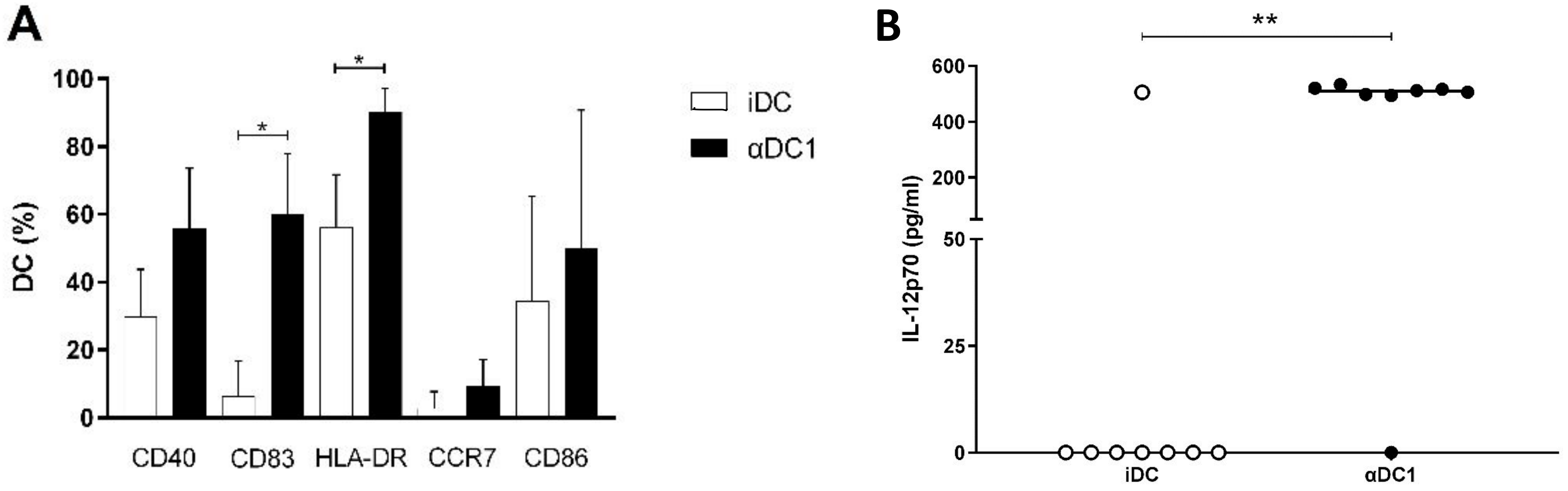

A
DC (%)
100
80
60
40
20
0
*
*
CD40
CD83
HLA-DR
CCR7
CD86
iDC
αDC1
B
**
IL-12p70 (pg/ml)
600
400
200
50
25
0
iDC
αDC1
